# Quantification of the smoking-associated cancer risk with rate advancement periods: meta-analysis of individual participant data from cohorts of the CHANCES consortium

**DOI:** 10.1186/s12916-016-0607-5

**Published:** 2016-04-05

**Authors:** José Manuel Ordóñez-Mena, Ben Schöttker, Ute Mons, Mazda Jenab, Heinz Freisling, Bas Bueno-de-Mesquita, Mark G. O’Doherty, Angela Scott, Frank Kee, Bruno H. Stricker, Albert Hofman, Catherine E. de Keyser, Rikje Ruiter, Stefan Söderberg, Pekka Jousilahti, Kari Kuulasmaa, Neal D. Freedman, Tom Wilsgaard, Lisette CPGM de Groot, Ellen Kampman, Niclas Håkansson, Nicola Orsini, Alicja Wolk, Lena Maria Nilsson, Anne Tjønneland, Andrzej Pająk, Sofia Malyutina, Růžena Kubínová, Abdonas Tamosiunas, Martin Bobak, Michail Katsoulis, Philippos Orfanos, Paolo Boffetta, Antonia Trichopoulou, Hermann Brenner, on behalf of the Consortium on Health and Ageing: Network of Cohorts in Europe and the United States (CHANCES)

**Affiliations:** Network Aging Research (NAR), Heidelberg University, Heidelberg, Germany; Division of Clinical Epidemiology and Aging Research, German Cancer Research Center (DKFZ), Im Neuenheimer Feld 581, D-69120 Heidelberg, Germany; International Agency for Research on Cancer (IARC), Lyon, France; Department of Chronic Diseases, National Institute for Public Health and the Environment (RIVM), Bilthoven, The Netherlands; Department of Gastroenterology and Hepatology, University Medical Centre, Utrecht, The Netherlands; Division of Epidemiology and Biostatistics, the School of Public Health, Imperial College London, London, United Kingdom; Department of Social & Preventive Medicine, Faculty of Medicine, University of Malaya, Kuala Lumpur, Malaysia; UKCRC Centre of Excellence for Public Health, Queens University of Belfast, Belfast, UK; Department of Epidemiology, Erasmus Medical Center, Rotterdam, The Netherlands; Department of Public Health and Clinical Medicine, Cardiology, and Heart Center, Umeå University, Umeå, Sweden; National Institute for Health and Welfare (THL), Helsinki, Finland; Nutritional Epidemiology Branch, Division of Cancer Epidemiology & Genetics, National Cancer Institute, Rockville, MD USA; Department of Community Medicine, UiT The Arctic University of Norway, Tromsø, Norway; Division of Human Nutrition, Wageningen University, Wageningen, The Netherlands; Institute of Environmental Medicine, Karolinska Institutet, Stockholm, Sweden; Nutritional Research, Department of Public Health and Clinical Medicine, and Arcum, Arctic Research Centre at Umeå University, Umeå, Sweden; Diet, Genes and Environment, Danish Cancer Society Research Center, Copenhagen, Denmark; Jagiellonian University Medical College, Faculty of Health Sciences, Krakow, Poland; Institute of Internal and Preventive Medicine, Novosibirsk, Russia; National Institute of Public Health, Prague, Czech Republic; Institute of Cardiology of Lithuanian University of Health Sciences, Kaunas, Lithuania; Department Epidemiology and Public Health, University College London, London, UK; Hellenic Health Foundation, Athens, Greece; University of Athens, Medical School, Department of Hygiene, Epidemiology and Medical Statistics, Athens, Greece; Institute for Translational Epidemiology and Tisch Cancer Institute, Icahn School of Medicine at Mount Sinai, New York, NY USA; German Cancer Consortium (DKTK), German Cancer Research Center (DKFZ), Heidelberg, Germany; Division of Preventive Oncology, German Cancer Research Center (DKFZ), Heidelberg, Germany

**Keywords:** Smoking, Cancer, Incidence, Mortality, Cohort, Meta-analysis

## Abstract

**Background:**

Smoking is the most important individual risk factor for many cancer sites but its association with breast and prostate cancer is not entirely clear. Rate advancement periods (RAPs) may enhance communication of smoking related risk to the general population. Thus, we estimated RAPs for the association of smoking exposure (smoking status, time since smoking cessation, smoking intensity, and duration) with total and site-specific (lung, breast, colorectal, prostate, gastric, head and neck, and pancreatic) cancer incidence and mortality.

**Methods:**

This is a meta-analysis of 19 population-based prospective cohort studies with individual participant data for 897,021 European and American adults. For each cohort we calculated hazard ratios (HRs) for the association of smoking exposure with cancer outcomes using Cox regression adjusted for a common set of the most important potential confounding variables. RAPs (in years) were calculated as the ratio of the logarithms of the HRs for a given smoking exposure variable and age. Meta-analyses were employed to summarize cohort-specific HRs and RAPs.

**Results:**

Overall, 140,205 subjects had a first incident cancer, and 53,164 died from cancer, during an average follow-up of 12 years. Current smoking advanced the overall risk of developing and dying from cancer by eight and ten years, respectively, compared with never smokers. The greatest advancements in cancer risk and mortality were seen for lung cancer and the least for breast cancer. Smoking cessation was statistically significantly associated with delays in the risk of cancer development and mortality compared with continued smoking.

**Conclusions:**

This investigation shows that smoking, even among older adults, considerably advances, and cessation delays, the risk of developing and dying from cancer. These findings may be helpful in more effectively communicating the harmful effects of smoking and the beneficial effect of smoking cessation.

**Electronic supplementary material:**

The online version of this article (doi:10.1186/s12916-016-0607-5) contains supplementary material, which is available to authorized users.

## Background

Although the global age-standardized smoking prevalence has decreased over the last 30 years, absolute numbers of smokers have increased with nearly one billion daily smokers worldwide in 2012 [1]. Today, smoking is a well-established risk factor for many common cancers [2–10]. However, associations with breast and prostate cancer are still a matter of debate [11–13]. The findings for these outcomes are often summarized with meta-analyses of published aggregate data. These are often subject to limitations regarding the estimations and conversions performed on the published data, the lack of or the variability of confounder adjustment between studies, the heterogeneity in the methodology employed, the variability of available data, and the populations included. Conducting meta-analyses of individual participant data would overcome such methodological shortcomings [14].

Standard epidemiological risk estimates, such as risk ratios, may not always be easily understood by the general population [15, 16] and might fail to properly communicate the harmful impact of smoking on cancer development and mortality. Rate advancement periods (RAPs) are designed to enhance quantification and communication of the harmful effect of smoking and the beneficial effect of quitting [17–19]. Thus, if the age at which a given level of cancer risk is reached is 65 years for never smokers and 55 years for current smokers, the RAP for current smoking would be 10 years, as the time would be advanced (or shortened) by this amount. Furthermore, if the age for that given level of risk is 59 years for those who have quit smoking for a defined time (e.g., 10-19 years) the RAP for quitting, expressed relative to current smoking, would be -4 years, as the time would be delayed by 4 years. Moreover, for cancers with available effective screening measures, RAPs may also provide useful information for a possible adaptation of the age at initiation of screening.

Therefore, we sought to quantify for the first time with RAPs the association of smoking exposure with total and site-specific cancer incidence and mortality using data from 19 population-based cohorts participating in the Consortium on Health and Aging: Network of Cohorts in Europe and the United States (CHANCES).

## Methods

### Study population

CHANCES is a coordinated multi-country study which aims at facilitating the harmonization of data from on-going prospective cohort studies in Europe and the USA in order to produce evidence on aging-related health characteristics and on determinants of healthy aging among the elderly in these countries (www.chancesfp7.eu) [20]. From all available participating studies in CHANCES, a total of 897,021 participants from 19 cohorts with cancer incidence/mortality data and smoking characteristics were included. Additional file 1: Table S1 provides an overview of the most important cohort characteristics. More detailed descriptions of included cohorts are openly available on the internet [21]. All included CHANCES cohorts obtained informed consent from all participants and were conducted according to the declaration of Helsinki.

### Definition of endpoints

Vital status and information on the cause of death was obtained from regional or state registries for all cohorts. Cancer incidence was ascertained by active follow-up or record linkage with national/regional cancer registries for most cohorts, except the HAPIEE cohorts and the SENECA study in which follow-up for cancer incidence was not performed. The main endpoints were total cancer incidence and mortality, as defined by codes C00-C97 according to the 10th edition of the International Classification of Diseases (ICD-10). Additional endpoints were incidence and mortality of the most frequent cancers in the CHANCES data that are known to be related to smoking, i.e., head and neck (C00-16, C30-32), gastric (C16), colon and rectum (C18 -20), pancreatic (C25), and lung cancer (C34), as well as cancers for which an association with smoking has not yet been established, i.e., breast (C50) and prostate cancer (C61).

### Smoking exposure assessment

Smoking status, categorized as never (reference), former, and current smoker was the main exposure and was available for all studies. Time since smoking cessation (≤9, 10 to 19, and ≥ 20 years ago) was available for all studies except for the Rotterdam study. Additionally, information on time since smoking cessation could not be harmonized for NIH-AARP and MORGAM FI studies. Current smokers were set as reference for the analyses of cancer risk with respect to time since smoking cessation. Smoking intensity (≤9, 10 to 19, or ≥ 20 smoked cigarettes per day) was available for all cohorts except SENECA. Duration of smoking (≤19, 20 to 39, or ≥ 40 years) was available for all cohorts but NIH-AARP.

### Statistical analyses

For analyses with cancer mortality outcomes, we included all participants with complete information on smoking status and vital status [*n* = 856,039 (95 %)]. For the analyses with cancer incidence outcomes, we only included participants without a prevalent cancer [*n* = 803,910 (90 %)]. Participants with missing values for the covariates included in the multivariable model [*n* = 76,441 (9 %)] were excluded from the analysis. Other approaches to deal with missing values, such as multiple imputation, may lead to bias [22] and do not increase precision substantially when missing data are less than 10 % [23].

Cox proportional hazard models were used to estimate hazard ratios (HR) and 95 % confidence intervals (CI) for the association of smoking exposure with cancer outcomes. We created two different models: one adjusted for age and sex only; and a multivariable model that included the most important common potential confounder variables for all endpoints that were also common to all included cohorts, i.e., age (continuous, years), sex, education (primary or less, more than primary but less than university or college, and university or college), vigorous physical activity (yes, no), history of diabetes (yes, no), BMI (continuous, kg/m^2^), and daily alcohol intake (continuous, g/day). In MORGAM Finland and Sweden cohorts physical activity was not available and therefore not adjusted for.

RAPs for a given smoking exposure variable (“smok_expo”) were calculated from the HRs for that given smoking exposure variable and the HR for age obtained in the Cox models by applying the following formula: RAP_smok_expo_ = (log HR_smok_expo_)/(log HR_age_). The calculation of their 95 % confidence intervals has been described in more detail elsewhere [24]. This calculation of the RAPs assumes that the risk of the disease exponentially increases with age, which is a fair assumption for cancer and is commonly made in Cox regression models including age as a linear term.

For both HRs and RAPs, sex- and age-stratified (younger or older than 65 years) analyses were conducted. Risk estimates for each cohort were derived from the individual participant data using a common analysis-script that runs in SAS, version 9.3 (Cary, NC, USA).

In order to allow for the variability of the true effect size between cohorts, meta-analyses with random effects models were used to derive summary HRs and RAPs [25]. Heterogeneity was tested for significance using Cochran’s Q test and quantified by the *I*^*2*^ estimate [26]. It was regarded as negligible if not significant (*P* < 0.05) or *I*^*2*^ < 30 %. Otherwise, if significant (*P* < 0.05), it was classified as moderate (30 % < *I*^*2*^ < 50 %), substantial (50 % < *I*^*2*^ < 75 %), or considerable (*I*^*2*^ > 75 %). When the heterogeneity was considerable, stratification of the meta-analyses according to cohort characteristics was carried out in order to examine possible sources of heterogeneity. Meta-analyses and tests of heterogeneity were derived in Microsoft Excel 2010 (Redmond, WA, USA) using the formulas described by Borenstein et al*.* [27]. Dose-response relations were assessed by meta-analysis for dose-response data using the Greenland and Longnecker method [28] and a random effects model as described elsewhere [29]. All statistical tests were two-sided using an alpha level of 0.05. This report was prepared in accordance with the PRISMA guidelines for the reporting of meta-analyses with individual participant data studies [30].

## Results

Socio-demographic and lifestyle characteristics of the participants at baseline across cohorts are shown in Table 1. The average age of participants was 60 years (ranging from 46 to 74 years). The proportion of men was similar in most cohorts, except for cohorts of men (COSM and MORGAM NI) or women (EPIC-Elderly NL and SMC). Despite variation across cohorts, the majority of participants were overweight, had an education under the university level (except NIH-AARP), and low consumption of alcohol. In total, 346,559 (39 %) participants were never smokers, 368,808 (41 %) former smokers, and 140,672 (16 %) current smokers.

**Table 1:** Socio-demographic and lifestyle characteristics at baseline of the participants included for cohorts in the CHANCES consortium

	COSM	EPIC-Elderly				ESTHER	HAPIEE				MORGAM			NIH-AARP	RS	SENECA	SMC	TROMSØ	VIP
		DK	ES	GR	NL		CZ	LT	PO	RU	FI	NI	SE						
*N* total	45,906	15,355	5185	9863	6896	9949	8857	7161	10,728	9360	38,108	2745	5476	566,279	8121	2585	38,984	10,463	95,000
Follow-up (years)^a^	13	13	13	10	13	11	8	4	7	6	17	16	11	12	12	8	13	13	14
Age (years)^a^	59	63	62	67	64	63	59	62	57	58	46	54	53	63	69	74	61	62	50
Sex, %																			
Male	100	46	43	40	5	45	47	45	49	46	48	100	49	60	39	50	0	47	50
Female	0	54	57	60	95	55	53	55	51	54	52	0	51	40	61	50	100	53	50
BMI (kg/m^2^)^a^	25	26	29	29	26	27	28	29	28	28	26	26	27	26	26	27	24	26	25
Education, %																			
Primary	70	41	87	91	34	75	13	7	12	10	46	1	31	1	25	67	74	56	23
Secondary	14	43	7	6	55	20	74	37	60	61	44	88	49	26	62	25	7	28	51
University	16	16	6	3	11	5	14	56	29	29	9	11	20	73	8	8	18	16	26
Alcohol (g/day)^a^	10	12	1	1	2	4	6	0	0	0	2	9	2	2	3	3	3	2	3
Vigorous physical activity, %																			
Yes	34	72	5	21	58	42	73	61	73	40	n.a.	12	n.a.	46	85	13	30	32	34
No	66	28	95	79	42	58	27	39	27	60		88		54	15	87	70	68	66
History of diabetes, %																			
Yes	7	3	11	14	5	16	12	8	12	5	5	2	5	9	7	9	5	4	2
No	93	97	89	86	95	84	88	92	88	95	95	98	95	91	93	91	95	96	98
Smoking status, %																			
Never	36	31	67	70	47	50	44	63	40	58	46	38	48	36	36	54	54	33	55
Former	39	36	16	19	35	33	30	18	28	14	29	33	32	51	41	28	23	36	25
Current	25	33	17	12	18	17	26	19	32	28	25	29	20	12	23	18	23	31	20
Time since smoking cessation, %																			
≤9 years	22	28	41	36	26	23	30	34	41	38	n.a.	36	38	26	n.a.	39	28	31	37
10–19 years	29	22	30	31	29	28	29	23	29	26		35	26	74		30	28	24	32
≥20 years	49	41	27	30	43	48	36	43	24	33		29	34			28	44	45	24

^a^The values shown are the mean for follow-up years and the median for age, BMI, and alcohol consumption

**Abbreviations** (alphabetically ordered): *BMI* body mass index, *COSM* Cohort of Swedish Men, *CZ* Czech Republic, *DK* Denmark, *EPIC* European Prospective Investigation into Cancer and Nutrition***,*** *ES* Spain, *ESTHER* Epidemiologische Studie zu Chancen der Verhütung, Früherkennung und optimierten Therapie chronischer Erkrankungen in der älteren Bevölkerung (German), *GR* Greece. *HAPIEE* Health, Alcohol and Psychosocial factors In Eastern Europe, *LT* Lithuania, *MORGAM* Monica Risk, Genetics, Archiving and Monograph, which included the cohorts *MORGAM FI* FINRISK Study (Finland), *MORGAM NI* PRIME Belfast Study (Northern Ireland), and *MORGAM SE* Northern Sweden Study (Norrbotten county only), *NIH-AARP* National Institute of Health – American Association of Retired Persons, *NL* the Netherlands, *PO* Poland, *RS* Rotterdam Study, *RU* Russia, *SENECA* Survey in Europe on Nutrition and the Elderly a Concerned Action, *SMC* Swedish Mammography Cohort, *VIP* Västerbotten Intervention Programme

### Association of smoking exposure with total and respiratory tract cancer incidence and mortality

The differences in risk estimates between the model adjusted only for age and sex and the multivariable model were lower than 10 % (data not shown); thus, only the results for the multivariable model are reported in detail. Smoking status was associated with increasing total, lung, and head and neck cancer incidence and mortality (Table 2). RAPs for current smokers ranged from 7.9 to 30.0 years and were stronger for cancer mortality than incidence outcomes, with the exception of lung cancer. Longer time since smoking cessation was associated with decreasing cancer incidence and mortality, with largest risk reductions for lung cancer followed by head and neck cancer and lastly total cancer. Higher smoking intensity and duration were associated with larger advancements in total, lung, and head and neck cancer risk and mortality (Additional file 2: Table S2).

**Table 2:** Associations of smoking status and time since smoking cessation with total, lung, head and neck cancer incidence and mortality^a,b^

Cancer site	Smoking exposure	Cancer incidence				Cancer mortality			
		Total^c^	Cases	HR (95 % CI)	RAP (95 % CI)	Total^c^	Cases	HR (95 % CI)	RAP (95 % CI)
Total cancer	Smoking status								
	Never	321984	43449	1.00 (reference)	0.00 (reference)	346559	13398	1.00 (reference)	0.00 (reference)
	Former	353311	64797	**1.15 (1.09 ; 1.21)** ^***^	**2.67 (1.65 ; 3.70)** ^**^	368808	24365	**1.39 (1.26 ; 1.54)** ^***^	**4.03 (2.85 ; 5.22)** ^***^
	Current	128615	26007	**1.44 (1.28 ; 1.63)** ^***^	**7.92 (5.58 ; 10.3)** ^***^	140672	13450	**2.19 (1.83 ; 2.63)** ^***^	**9.92 (7.84 ; 12.0)** ^***^
	Years since smoking cessation (reference: current smokers)^d^								
	≤9 years	19049	2704	**0.90 (0.86 ; 0.94)**	**-1.62 (-2.41; -0.83)**	22693	1351	**0.83 (0.77 ; 0.89)**	**-2.09 (-2.86 ; -1.31)**
	10–19 years	18511	2613	**0.80 (0.74 ; 0.88)** ^**^	**-4.01 (-5.73; -2.29)** ^**^	21361	1145	**0.66 (0.59 ; 0.73)**	**-4.81 (-6.01 ; -3.62)**
	≥20 years	24651	3904	**0.75 (0.70 ; 0.81)** ^**^	**-5.27 (-6.69; -3.86)** ^*^	28057	1507	**0.52 (0.47 ; 0.58)** ^*^	**-7.54 (-8.59 ; -6.49)**
	P linear trend			**<0.0001**				**<0.0001**	
Lung cancer	Smoking status								
	Never	321984	923	1.00 (reference)	0.00 (reference)	346559	863	1.00 (reference)	0.00 (reference)
	Former	353311	6785	**4.06 (3.13 ; 5.26)** ^**^	**16.4 (12.2 ; 20.7)** ^***^	368808	6967	**4.10 (3.14 ; 5.36)** ^***^	**15.3 (11.7; 18.9)** ^**^
	Current	128615	6333	**13.1 (9.90 ; 17.3)** ^***^	**30.0 (24.1 ; 35.9)** ^***^	140672	6165	**11.5 (8.21 ; 16.1)** ^***^	**26.2 (21.5; 30.8)** ^***^
	Years since smoking cessation (reference: current smokers)^d^								
	≤9 years	19049	306	**0.60 (0.48 ; 0.73)** ^*^	**-5.26 (-7.91; -2.61)** ^*^	22693	373	**0.70 (0.56 ; 0.87)** ^**^	**-3.45 (-5.33 ; -1.56)**
	10–19 years	18511	191	**0.33 (0.25 ; 0.44)** ^**^	**-12.3 (-16.4; -8.20)** ^**^	21361	233	**0.40 (0.31 ; 0.51)** ^*^	**-8.99 (-12.2 ; -5.77)** ^**^
	≥20 years	24651	139	**0.15 (0.12 ; 0.19)**	**-21.9 (-28.1; -15.8)** ^**^	28057	168	**0.18 (0.14 ; 0.24)** ^*^	**-17.0 (-21.3 ; -12.7)** ^**^
	P linear trend			**<0.0001**				**<0.0001**	
Head and neck cancer	Smoking status								
	Never	321984	636	1.00 (reference)	0.00 (reference)	346559	155	1.00 (reference)	0.00 (reference)
	Former	353311	1503	**1.73 (1.57 ; 1.92)**	**7.77 (4.24 ; 11.3)**	368808	388	**2.10 (1.70 ; 2.61)**	**9.01 (4.36 ; 13.6)**
	Current	128615	1051	**2.89 (1.98 ; 4.21)** ^**^	9.10 (-2.34 ; 20.5)^***^	140672	359	**3.74 (2.38 ; 5.89)**	**14.0 (4.53 ; 23.5)** ^**^
	Years since smoking cessation (reference: current smokers)^d^								
	≤9 years	19049	64	1.08 (0.80 ; 1.47)	-0.77 (-3.97 ; 3.66)	22693	22	1.35 (0.75 ; 2.44)	-2.64 (-4.11 ; 9.40)
	10–19 years	18511	33	**0.61 (0.40 ; 0.92)**	-5.71 (-15.4 ; 1.19)	21361	14	1.35 (0.62 ; 2.90)	3.45 (-7.93 ; 14.8)
	≥20 years	24651	53	**0.55 (0.34 ; 0.91)**	-2.75 (-9.26 ; 3.76)	28057	20	0.58 (0.31 ; 1.07)	-3.59 (-10.1 ; 2.91)
	P linear trend			**0.0039**				0.0676	

^a^Numbers in bold denote statistical significance (*P* < 0.05). Heterogeneity was regarded as negligible if not significant (*P* < 0.05) or *I* ^*2*^ < 30 %. Otherwise, if significant (*P* < 0.05), it was classified as ^*^ moderate (30 % < *I* ^*2*^ < 50 %), ^**^ substantial (50 % < *I* ^*2*^ < 75 %), or ^***^ considerable (*I* ^*2*^ > 75 %)

^b^Cohort-specific Hazard Ratios (HRs) and Rate Advancement Periods (RAPs) were summarized with meta-analyses using random effects models. HRs and RAPs were adjusted for sex, age, BMI, education, vigorous physical activity, history of diabetes, and alcohol consumption

^c^The total number of participants for the analyses with cancer incidence is smaller because the participants with a diagnosis of cancer before baseline were excluded. Furthermore, some cohorts (HAPIEE and SENECA cohorts) had no cancer incidence data available for the analyses

^d^For the analyses with the categories of years since smoking cessation, the data from the NIH-AARP and MORGAM FI were not included because of the different categories employed

*HAPIEE* Health, Alcohol and Psychosocial Factors in Eastern Europe, *SENECA* Survey in Europe on Nutrition and the Elderly a Concerned Action, *NIH-AARP* National Institute of Health – American Association of Retired Persons, *MORGAM* Monica Risk, Genetics, Archiving and Monograph, which included the cohort *MORGAM FI* FINRISK Study (Finland)

Overall, considerable heterogeneity between studies was observed (*I*^*2*^ > 75 %), particularly for total and lung cancer outcomes. Risk estimates were largest in the United States, followed by Eastern Europe, and then by other regions of Europe (Additional files 3 and 4: Tables S3 and S4 for total and lung cancer, respectively). Larger effects were seen with shorter follow-ups, more recent initiation of the study, and among studies with lower numbers of cases.

### Association of smoking exposure with digestive tract cancer incidence and mortality

Smoking status was also associated with higher colorectal, gastric, and pancreatic cancer incidence and mortality (Table 3). RAPs for colorectal, gastric, and pancreatic cancer incidence were similar to those for mortality. Being a current smoker (compared with never smoking) significantly advanced the risk of developing colorectal, gastric, and pancreatic cancer by 3.6, 5.6, and 7.6 years, respectively. Quitting smoking (compared with not quitting) significantly delayed the risk of development of, and death from, colorectal (up to 3.2 years), gastric (up to 5.6 years), and pancreatic cancer (up to 10.4 years). Higher smoking intensity and duration were in most cases associated with larger advancements in digestive tract cancer risk and mortality (Additional file 5: Table S5). The degree of heterogeneity between studies in the meta-analyses was mostly negligible (*P* > 0.05 and *I*^*2*^ < 30 %).

**Table 3:** Associations of smoking status and time since smoking cessation with colorectal, gastric and pancreatic cancer incidence and mortality^a,b^

Cancer site	Smoking exposure	Cancer incidence				Cancer mortality			
		Total^c^	Cases	HR (95 % CI)	RAP (95 % CI)	Total^c^	Cases	HR (95 % CI)	RAP (95 % CI)
Colorectal cancer	Smoking status								
	Never	321984	4359	1.00 (reference)	0.00 (reference)	346559	1702	1.00 (reference)	0.00 (reference)
	Former	353311	6273	**1.20 (1.15 ; 1.25)**	**2.62 (2.00 ; 3.24)**	368808	2264	**1.22 (1.13 ; 1.31)**	**2.19 (1.35 ; 3.02)**
	Current	128615	2064	**1.20 (1.07 ; 1.34)** ^*^	**3.64 (2.81 ; 4.46)**	140672	912	**1.35 (1.16 ; 1.58)**	**4.61 (3.53 ; 5.68)**
	Years since smoking cessation (reference: current smokers)^d^								
	≤9 years	19049	318	1.00 (0.87 ; 1.16)	-0.11 (-1.94 ; 1.72)	22693	152	1.07 (0.86 ; 1.32)	0.22 (-2.09 ; 2.53)
	10–19 years	18511	365	1.11 (0.97 ; 1.27)	1.16 (-0.53 ; 2.84)	21361	167	1.07 (0.87 ; 1.31)	0.31 (-1.83 ; 2.45)
	≥20 years	24651	514	0.88 (0.78 ; 1.00)	**-1.95 (-3.58 ; -0.32)**	28057	205	**0.76 (0.63 ; 0.93)**	**-3.18 (-5.24 ; -1.11)**
	P linear trend			0.1885				**0.0134**	
Gastric cancer	Smoking status								
	Never	321984	598	1.00 (reference)	0.00 (reference)	346559	463	1.00 (reference)	0.00 (reference)
	Former	353311	880	1.18 (0.95; 1.46)	1.80 (-0.31 ; 3.91)	368808	631	**1.31 (1.02 ; 1.68)**	**2.08 (0.02 ; 4.14)**
	Current	128615	388	**1.74 (1.50; 2.02)**	**5.62 (3.85 ; 7.39)**	140672	302	**1.73 (1.36 ; 2.19)**	**5.22 (3.08 ; 7.36)**
	Years since smoking cessation (reference: current smokers)^d^								
	≤9 years	19049	54	0.85 (0.60 ; 1.20)	-3.02 (-6.43 ; 0.40)	22693	61	1.13 (0.80 ; 1.58)	-0.59 (-3.90 ; 2.72)
	10–19 years	18511	51	0.68 (0.41 ; 1.12)	-3.48 (-7.00 ; 0.05)	21361	45	0.72 (0.46 ; 1.14)	-2.62 (-6.57 ; 1.32)
	≥20 years	24651	77	**0.69 (0.51 ; 0.93)**	-2.42 (-5.08 ; 0.24)	28057	77	0.87 (0.64 ; 1.19)	-1.89 (-5.25 ; 1.47)
	P linear trend			**0.0461**				0.2355	
Pancreatic cancer	Smoking status								
	Never	321984	921	1.00 (reference)	0.00 (reference)	346559	1186	1.00 (reference)	0.00 (reference)
	Former	353311	1216	1.13 (0.95 ; 1.35)	1.45 (0.23 ; 2.67)	368808	1609	1.19 (0.98 ; 1.45)	1.85 (0.85 ; 2.86)
	Current	128615	635	**1.90 (1.48 ; 2.43)** ^*^	**7.57 (4.31 ; 10.8)** ^*^	140672	808	**2.19 (1.74 ; 2.75)** ^**^	**8.50 (6.45 ; 10.5)**
	Years since smoking cessation (reference: current smokers)^d^								
	≤9 years	19049	74	0.83 (0.62 ; 1.11)	-2.16 (-6.01 ; 1.69)	22693	93	**0.72 (0.56 ; 0.93)**	**-3.78 (-6.73 ; -0.84)**
	10–19 years	18511	62	**0.71 (0.52 ; 0.96)**	**-4.82 (-9.11 ; -0.53)**	21361	81	**0.63 (0.48 ; 0.82)**	**-5.57 (-8.74 ; -2.40)**
	≥20 years	24651	65	**0.47 (0.31 ; 0.70)**	**-9.72 (-15.3 ; -4.15)**	28057	104	**0.48 (0.35 ; 0.67)**	**-10.4 (-13.7 ; -7.16)**
	P linear trend			**<0.0001**				**<0.0001**	

^a^Numbers in bold denote statistical significance (*P* < 0.05). Heterogeneity was regarded as negligible if not significant (*P* < 0.05) or *I* ^*2*^ < 30 %. Otherwise, if significant (*P* < 0.05), it was classified as ^*^ moderate (30 % < *I* ^*2*^ < 50 %), ^**^ substantial (50 % < *I* ^*2*^ < 75 %), or ^***^ considerable (*I* ^*2*^ > 75 %)

^b^Cohort-specific Hazard Ratios (HRs) and Rate Advancement Periods (RAPs) were summarized with meta-analyses using random effects models. HRs and RAPs were adjusted for sex, age, BMI, education, vigorous physical activity, history of diabetes and alcohol consumption

^c^The total number of participants for the analyses with cancer incidence is smaller because the participants with a diagnosis of cancer before baseline were excluded. Furthermore, some cohorts (HAPIEE and SENECA cohorts) had no cancer incidence data available for the analyses

^d^For the analyses with the categories of years since smoking cessation, the data from the NIH-AARP and MORGAM FI were not included because they had different categories available

*HAPIEE* Health, Alcohol and Psychosocial Factors in Eastern Europe, *SENECA* Survey in Europe on Nutrition and the Elderly a Concerned Action, *NIH-AARP* National Institute of Health – American Association of Retired Persons, *MORGAM* Monica Risk, Genetics, Archiving and Monograph, which included the cohort *MORGAM FI* FINRISK Study (Finland)

### Association of smoking exposure with sex-specific cancer incidence and mortality

Smoking status was significantly associated with moderate increases in breast cancer incidence and mortality, although RAPs suggested larger advancements in the risk of both outcomes (Table 4). Smoking intensity was furthermore tentatively associated with breast cancer incidence and more strongly associated with breast cancer mortality (Additional file 6: Table S6).

**Table 4:** Associations of smoking status and time since smoking cessation with sex-specific cancer incidence and mortality^a,b^

Cancer site	Smoking exposure	Cancer incidence				Cancer mortality			
		Total^c^	Cases	HR (95 % CI)	RAP (95 % CI)	Total^c^	Cases	HR (95 % CI)	RAP (95 % CI)
Breast cancer	Smoking status								
	Never	174507	7121	1.00 (reference)	0.00 (reference)	191907	1197	1.00 (reference)	0.00 (reference)
	Former	116656	5428	**1.08 (1.04 ; 1.12)**	**2.37 (0.68 ; 4.06)**	121725	905	**1.15 (1.05 ; 1.27)**	**2.71 (0.78 ; 4.63)**
	Current	59755	2536	**1.07 (1.00 ; 1.15)**	**3.83 (1.76 ; 5.91)**	64470	466	**1.28 (1.06 ; 1.55)**	**5.10 (2.47 ; 7.72)**
	Years since smoking cessation (reference: current smokers)^d^								
	≤9 years	8348	275	0.97 (0.84 ; 1.13)	-2.49 (-7.49 ; 2.52)	9726	49	0.98 (0.57 ; 1.67)	-1.82 (-8.21; 4.57)
	10–19 years	7044	253	1.03 (0.81 ; 1.31)	-3.87 (-9.84 ; 2.10)	8092	43	1.02 (0.70 ; 1.49)	0.51 (-7.55 ; 8.57)
	≥20 years	8437	333	1.03 (0.85 ; 1.24)	-3.77 (-10.2 ; 2.66)	9539	61	1.23 (0.69 ; 2.21)	-0.56 (-8.57 ; 1.48)
	P linear trend			0.7293				0.4549	
Prostate cancer	Smoking status								
	Never	147477	11090	1.00 (reference)	0.00 (reference)	154652	920	1.00 (reference)	0.00 (reference)
	Former	236655	17257	**0.88 (0.82 ; 0.95)** ^*^	**-1.67 (-2.80; -0.54)** ^**^	247083	1644	1.04 (0.94 ; 1.15)	0.29 (-0.33 ; 0.91)
	Current	68860	3701	**0.81 (0.72 ; 0.91)** ^**^	**-2.89 (-4.81; -0.97)** ^**^	76202	589	1.26 (0.97 ; 1.64)^**^	**1.88 (0.25 ; 3.51)**
	Years since smoking cessation (reference: current smokers)^d^								
	≤9 years	10701	536	1.00 (0.90 ; 1.12)	0.51 (-0.83 ; 1.84)	12967	98	0.94 (0.64 ; 1.37)	-1.03 (-3.35 ; 1.30)
	10–19 years	11467	702	1.03 (0.89 ; 1.19)	1.09 (-0.17 ; 2.35)	13269	130	0.95 (0.74 ; 1.20)	-0.43 (-2.18 ; 1.32)
	≥20 years	16214	1227	1.08 (0.99 ; 1.18)	0.75 (-0.38 ; 1.88)	18518	228	0.82 (0.67 ; 1.00)	**-1.71 (-3.18; -0.24)**
	P linear trend			**0.0480**				0.0838	

^a^Numbers in bold denote statistical significance (*P* < 0.05). Heterogeneity was regarded as negligible if not significant (*P* < 0.05) or *I* ^*2*^ < 30 %. Otherwise, if significant (*P* < 0.05), it was classified as ^*^ moderate (30 % < *I* ^*2*^ < 50 %), ^**^ substantial (50 % < *I* ^*2*^ < 75 %), or ^***^ considerable (*I* ^*2*^ > 75 %)

^b^Cohort-specific Hazard Ratios (HRs) and Rate Advancement Periods (RAPs) were summarized with meta-analyses using random effects models. HRs and RAPs were adjusted for sex, age, BMI, education, vigorous physical activity, history of diabetes, and alcohol consumption

^c^The total number of participants equals to the total number of women (for breast cancer) or men (for prostate cancer). The total number of participants for the analyses with cancer incidence is smaller because the participants with a diagnosis of cancer before baseline were excluded. Furthermore, some cohorts (HAPIEE and SENECA cohorts) had no cancer incidence data available for the analyses

^d^For the analyses with the categories of years since smoking cessation, the data from the NIH-AARP and MORGAM FI were not included because they had different categories available

*HAPIEE* Health, Alcohol and Psychosocial Factors in Eastern Europe, *SENECA* Survey in Europe on Nutrition and the Elderly a Concerned Action, *NIH-AARP* National Institute of Health – American Association of Retired Persons, *MORGAM* Monica Risk, Genetics, Archiving and Monograph, which included the cohort *MORGAM FI* FINRISK Study (Finland)

Smoking status was associated with lower prostate cancer incidence, but associated with higher prostate cancer mortality (although not reaching statistical significance). RAPs for current smokers suggested a 2.9 year delay in prostate cancer risk compared with never smokers; but an advancement of 1.9 years in the risk of dying from prostate cancer. Overall, time since smoking cessation was not significantly associated with prostate cancer outcomes, but a 1.7 year delay in the risk of dying from prostate cancer was observed among those who stopped smoking more than 20 years previously, compared with those who were still smokers at the initiation of the study. Smoking intensity was also inversely associated with prostate cancer incidence but associated with increased mortality (Additional file 6: Table S6).

### Sex- and age-stratified analyses of smoking exposure and cancer incidence and mortality

**Fig. 1:**
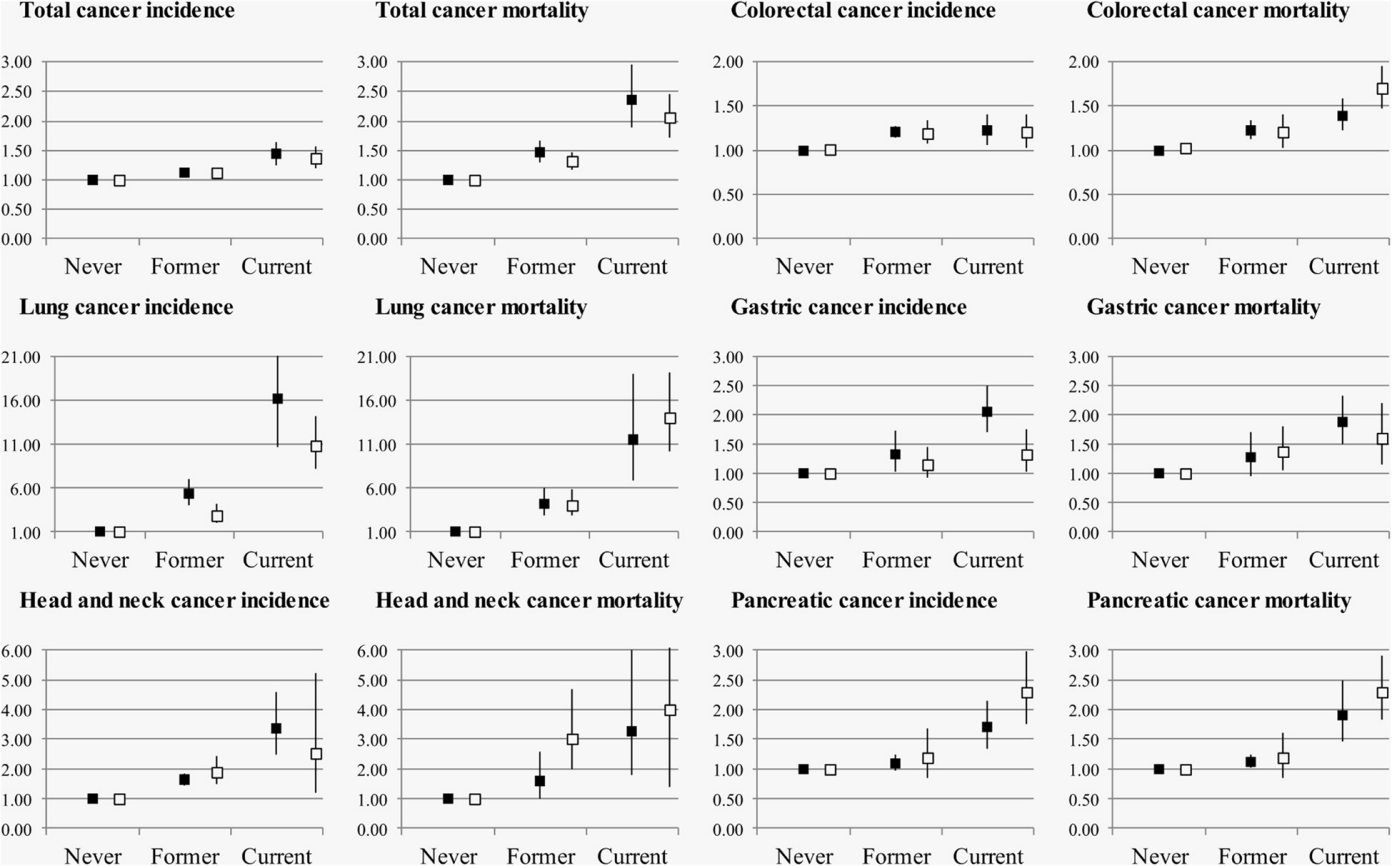
Sex-stratified association of smoking status with cancer incidence and mortality. Hazard ratios (HR) and 95 % confidence intervals (CI) for cancer incidence and mortality are depicted on the vertical axis for current and former smokers (never smokers as reference). Cohort-specific HRs and 95 % CIs were pooled with meta-analyses separately for men (*black squares*) and women (*white squares*)

**Fig. 2:**
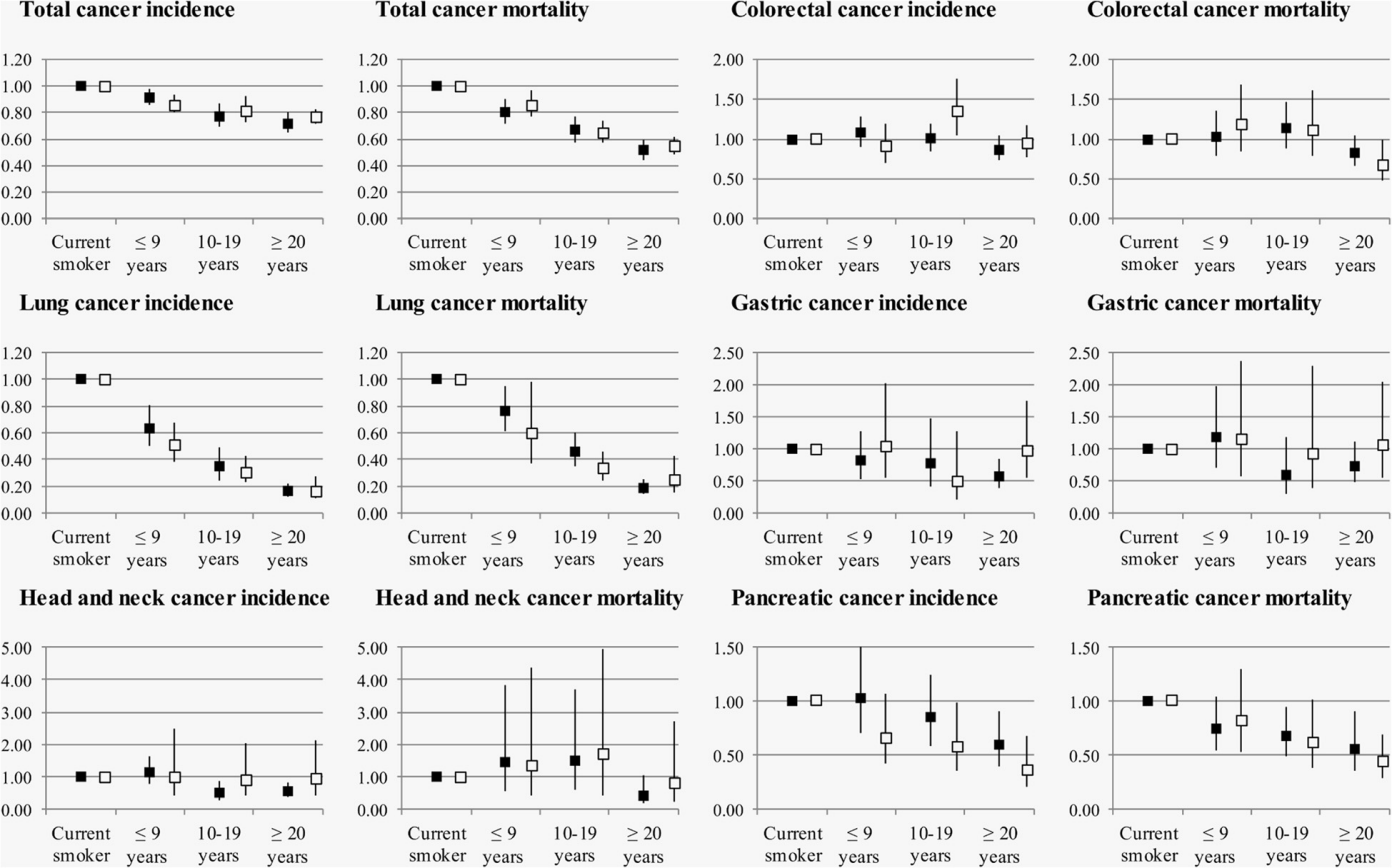
Sex-stratified association of time since smoking cessation with cancer incidence and mortality. Hazard ratios (HR) and 95 % confidence intervals (CI) for cancer incidence and mortality are depicted on the vertical axis for smoking cessation ≤ 9 years ago, 10–19 years ago, or ≥ 20 years ago (current smokers as reference). Cohort-specific HRs and 95 % CIs were pooled with meta-analyses separately for men (*black squares*) and women (*white squares*)

Overall, smoking status was associated with cancer incidence and mortality for all sites with few differences between men and women (Fig. 1). Only for lung and gastric cancer incidence, stronger risks were observed among former or current smoking men when compared with women. In both men and women, longer time since smoking cessation was associated with significant decreases in total, lung, and pancreatic cancer incidence and mortality (Fig. 2). RAPs were homogeneous among sexes.

**Fig. 3:**
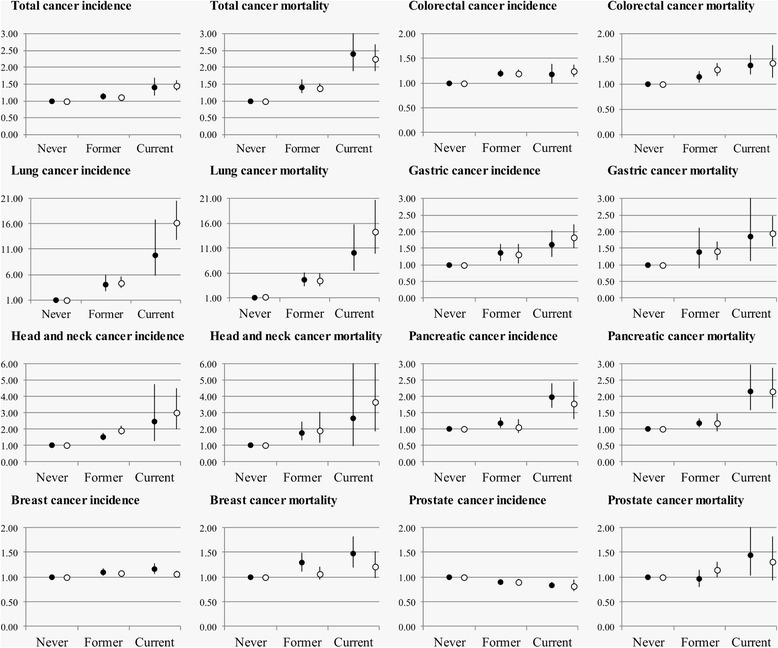
Age-stratified association of smoking status with cancer incidence and mortality. Hazard ratios (HR) and 95 % confidence intervals (CI) for cancer incidence and mortality are depicted on the vertical axis for current and former smokers (never smokers as reference). Cohort-specific HRs and 95 % CIs were pooled with meta-analyses separately for older than 65 years old (*black circles*) and younger than 65 years old (*white circles*)

**Fig. 4:**
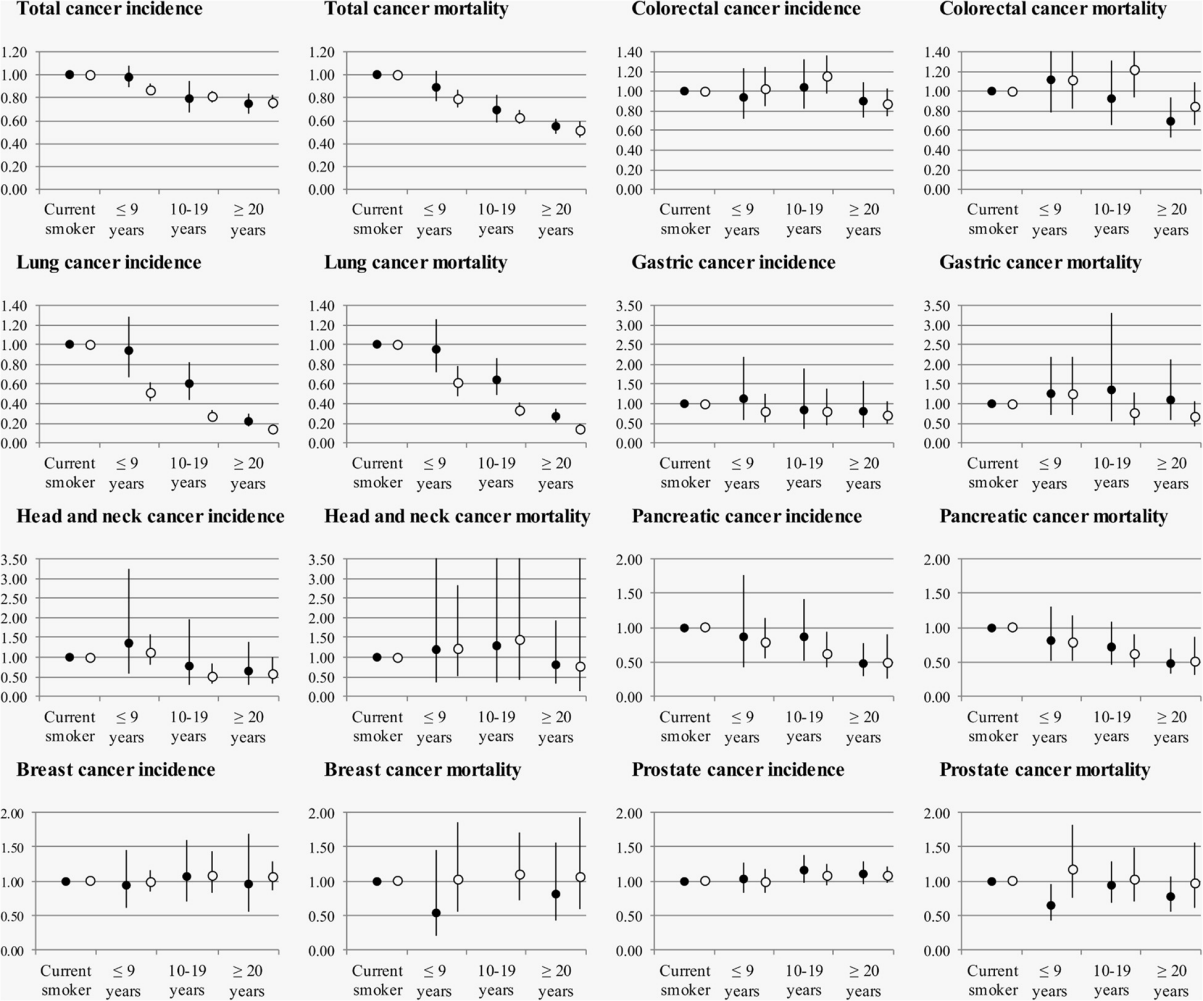
Age-stratified association of time since smoking cessation with cancer incidence and mortality. Hazard ratios (HR) and 95 % confidence intervals (CI) for cancer incidence and mortality are depicted on the vertical axis for smoking cessation ≤ 9 years ago, 10–19 years ago, or ≥ 20 years ago (current smokers as reference). Cohort-specific HRs and 95%CIs were pooled with meta-analyses separately for older than 65 years old (*black circles*) and younger than 65 years old (*white circles*)

Overall, the associations of smoking status and time since smoking cessation with cancer outcomes were similar for younger and older adults (under and above 65 years, respectively). Only for lung cancer incidence and mortality, a clearly larger relative increase in cancer risk among current smokers (Fig. 3), and a larger relative reduction in cancer risk with longer time since smoking cessation (Fig. 4) was observed among younger compared with older adults. The results with RAPs were homogeneous among age groups.

## Discussion

In this large individual participant meta-analysis of 19 cohort studies including data from 897,021 adults from Europe and the United States, we observed that current smoking advanced the risk of developing and dying from any cancer by almost 8 and 10 years, respectively, compared with never smokers. The benefit of quitting was observed both immediately and in the long run with as much as 2 years delay in the risk of developing and dying from cancer in those who quit only less than 10 years ago. This benefit increased to almost 8 years delay in the risk of cancer death among those who quit more than 20 years ago.

Overall, relative risk estimates for smoking were larger for cancer mortality than for cancer incidence. There may be two main explanations for this finding: first, smoking is more strongly associated with cancers that have a poor prognosis, such as lung cancer. Second, smoking also adversely affects prognosis after cancer diagnosis as underlined in the 2014 Report of the Surgeon General [2]. The association of smoking exposure with the different cancer sites greatly varied in magnitude, with HRs and RAPs being largest for lung, followed by head and neck, pancreatic, gastric, colorectal, and breast cancer, in this declining order. Inconsistent associations of smoking exposure with prostate cancer incidence and mortality were observed.

Large heterogeneity between studies was observed for total and lung cancer, the main contributor being the study location, with larger effect sizes observed in North America than in Europe. Such geographical difference has previously been described for lung cancer [4]. Other cohort characteristics, such as the year of initiation of the study and the length of follow-up, may have also contributed to the heterogeneity although to a lesser degree. In particular, stratification of our analyses by sex or age did not reduce the heterogeneity. For all other cancer sites, heterogeneity was largely negligible.

The magnitudes of the effects observed were comparable to those previously seen in the literature, especially for lung [4], head and neck [7], gastric [8], and pancreatic cancer [9, 10]. For colorectal, breast, and prostate cancer there has been more debate as to whether smoking is a true risk factor. We will therefore discuss these cancer sites in the following paragraphs.

Previous studies on colorectal cancer have mostly focused on the impact of smoking on incidence [5]. We provide substantial evidence that cigarette smoking increases colorectal cancer mortality. In previous meta-analyses, larger increases in risk among former smokers than current smokers were often observed suggesting a long lasting effect of smoking [5, 6]. Although in our study current smokers had increased colorectal cancer incidence and mortality, risk reductions were not visible for time since smoking cessation shorter than 20 years, which reinforces the before-mentioned suggestions. We also observed increased colorectal cancer incidence and mortality with greater smoking intensity and duration, which further suggests a causal role of smoking in colorectal cancer development. Furthermore, little to no variation by sex and age was observed, therefore suggesting that the impact of cigarette smoking and time since smoking cessation on colorectal cancer is independent of sex and age.

Previous studies have reported weak associations of tobacco smoking with breast cancer incidence which is in line with our findings [11, 31–33]. There has been debate as to the extent to which the effect of smoking on breast cancer incidence might be due to confounding by alcohol consumption [31–33]. While some have observed increased breast cancer risks associated with smoking among nondrinkers [31], and others among drinkers [32], a more recent analysis concluded that risk did not differ by alcohol consumption [33]. In our analyses we observed statistically significant advancements in the risk of breast cancer incidence and mortality among current and former smokers compared with never smokers, even after adjusting for alcohol consumption. However, no consistent associations with time since cessation, smoking intensity, and duration were observed.

Our finding that current smokers had lower prostate cancer incidence than never smokers is consistent with reports from previous studies [13, 34]. However, this apparent protective effect seems to be confined only to low-grade/localized prostate tumors, whereas higher-grade/advanced prostate tumors were directly associated with smoking [13]. We observed higher prostate cancer mortality among current smokers and an advancement of nearly 2 years of the risk of prostate cancer death among current smokers. We also observed a delay in the risk of prostate cancer mortality by nearly 2 years after 20 years since smoking cessation. Furthermore, both higher smoking intensity and duration were associated with increased prostate cancer mortality. A plausible explanation for the apparent differences between prostate cancer incidence and mortality may be that current smokers might be less likely to seek medical attention and undergo prostate cancer screening than never smokers, therefore being less often diagnosed with low-grade/localized tumors. Alternatively, mechanisms have been proposed by which cigarette smoking could protect against prostate cancer [13, 34].

Our main advantage was the availability of harmonized individual participant data for the estimation of cohort-specific risk estimates. This allowed us to define and use similar categories of exposure, similar disease endpoints, and common multivariable models among the included studies. Our investigation also included only prospective cohort studies, which reduces the potential of biases, often of concern in retrospective studies, such as recall and selection bias. Finally, due to the large sample size of our analyses we were able to assess the association of smoking exposure with cancer endpoints among older adults (>65 years) and thereby to provide specific evidence that the detrimental effects of smoking and the benefits of cessation prevail even at old age.

Our main limitation refers to the assessment of smoking status, which relies on the validity of the participants’ responses in the questionnaires. Since we only employed baseline data, some of the current smokers at baseline may have quit during follow-up, thus cancer risk among current smokers may have been underestimated. On the other hand, some quitters may have resumed smoking which could have led to an overestimation of cancer risk among former smokers. Although we adjusted for the most important common confounders, due to the lack of relevant covariates in some of the included cohorts, the possibility of residual confounding cannot be excluded. Dietary variables or family history of cancer have been related to smoking status [35–37]. However, due to their weak effects on cancer risk, their influence in the association of smoking with cancer is expected to be very small. Finally, despite our large sample size we could only focus on the most common cancer sites across all included cohorts.

## Conclusions

We showed that smoking increases cancer incidence and mortality in all sites (except for prostate cancer incidence) and that quitting smoking is still beneficial at old age. Lastly, although there have been other attempts to quantify the benefits of smoking cessation in years by which the excess in cancer risk is decreased [38, 39], we have shown for the first time with RAPs how smoking significantly advances the risk of developing and dying from major cancers, being a better communication tool than the concept of risk alone. Risk communication is especially crucial in promoting smoking cessation and RAPs could be easier to grasp for the general public considering the benefits of quitting. RAPs are certainly less susceptible to the sort of bias highlighted by Peto [40], whereby the fact that relative risks fall after quitting implies nothing about absolute risks (which grow more slowly). Given the higher susceptibility of older adults to the harmful effects of smoking and the lack of smoking-specific public health policies aimed at this group [41, 42], the results of this study underline the need for continued and enhanced efforts to decrease tobacco smoking prevalence in Europe and the United States.

### Ethics approval and consent to participate

The included studies have been approved by local ethics committees: COSM: Regional Ethical Review Board at Karolinska Institutet (Stockholm, Sweden); EPIC-Elderly: Ethics Committee of the International Agency for Research on Cancer and at each participating centre; EPIC-Elderly DK: The National Committee on Health Research Ethics; EPIC-Elderly ES: Comité de Ética de Investigación Clínica (CEIC); EPIC-Elderly GR: ethics committees of the University of Athens Medical School and the Hellenic Health Foundation; EPIC-Elderly NL: Institutional Review Board of the University Medical Center Utrecht and the Medical Ethical Committee of TNO Nutrition and Food Research; ESTHER: Medical Faculty of the University of Heidelberg and the Medical Association of Saarland; HAPIEE: University College London (Great Britain), National Institute of Public Health (Prague, Czech Republic), Jagiellonian University (Krakow, Poland), and Lithuanian University of Health Sciences (Kaunas, Lithuania); MORGAM FI: 1980s: no ethics approval required for observational studies (but current laws allow the use of these data for public health research), 1990s: Ethics committee of the National Public Health Institute (KTL), 2002: Ethics Committee of Epidemiology and Public Health in Hospital District of Helsinki and Uusimaa; MORGAM NI: Queen’s University of Belfast Ethical Committee (Belfast, Northern Ireland); MORGAM SE: Research Ethics Committee of Umeå University (Umeå, Sweden); NIH-AARP: Special Studies Institutional Review Board of the NCI; RES: Erasmus University Medical Centre (Rotterdam, the Netherlands); SENECA: Local ethics approval was obtained by the SENECA participating centres; SMC: Regional Ethical Board at Karolinska Institutet (Stockholm, Sweden); TROMSØ: Regional Committee for Medical and Health Research Ethics and the Data Inspectorate of Norway; VIP: Regional Ethical Review Board of Umeå University (Umeå, Sweden).

### Consent for publication

Informed consent has been obtained from all participants included in the analyzed studies, and the studies are being conducted in accordance with the declaration of Helsinki.

### Availability of data and materials

The CHANCES participating cohorts’ data are available only to the collaborating scientists from the respective CHANCES participating centers. The data may be available upon request for some of the participating centers but not for all due to relevant data protection laws.

## Additional files

Additional file 1: Main characteristics of cohorts participating in the current CHANCES investigation (Table S1). (DOC 56 kb) Additional file 2: Associations of smoking intensity and duration with total and respiratory (DOC 89 kb) Additional file 3: Stratification of meta-analyses and reassessment of heterogeneity for the association of smoking status with total cancer incidence and mortality according to the general cohort characteristics (Table S3). (DOC 82 kb) Additional file 4: Stratification of meta-analyses and reassessment of heterogeneity for the association of smoking status with lung cancer incidence and mortality according to the general cohort characteristics (Table S4). (DOC 79 kb) Additional file 5: Associations of smoking intensity and duration with total and digestive tract cancer incidence and mortality (Table S5). (DOC 88 kb) Additional file 6: Associations of smoking intensity and duration with sex-specific cancer incidence and mortality (Table S6). (DOC 70 kb)

## Abbreviations

BMI: Body mass index
CHANCES: Consortium on Health and Aging: Network of Cohorts in Europe and the United States
CI: Confidence interval
COSM: Cohort OF Swedish Men
CZ: Czech Republic
DK: Denmark
EPIC: European Prospective Investigation into Cancer and Nutrition
ES: Spain
ESTHER: Epidemiologische Studie zu Chancen der Verhütung, Früherkennung und optimierten Therapie chronischer Erkrankungen in der älteren Bevölkerung (German)
GR: Greece
HAPIEE: Health, Alcohol and Psychosocial Factors in Eastern Europe
HR: Hazard ratio
ICD: International Classification of Diseases
LT: Lithuania
MORGAM: Monica Risk, Genetics, Archiving and Monograph, which included the cohorts
MORGAM FI: FINRISK Study (Finland)
MORGAM NI: PRIME Belfast Study (Northern Ireland)
MORGAM SE: Northern Sweden Study (Norrbotten county only)
NIH-AARP: National Institute of Health – American Association of Retired Persons
NL: the Netherlands
PO: Poland
RAPs: Risk or rate advancement period
RS: Rotterdam Study
RU: Russia
SE: Sweden
SENECA: Survey in Europe on Nutrition and the Elderly a Concerned Action
SMC: Swedish Mammography Cohort
VIP: Västerbotten Intervention Programme
