# Supplementary material for: Quantification of the smoking-associated cancer risk with rate advancement periods: meta-analysis of individual participant data from cohorts of the CHANCES consortium
Source: BMC Med. 2016 Apr 5;14:62. doi: 10.1186/s12916-016-0607-5 (PMC4820956; doi:10.1186/s12916-016-0607-5)
Supplement: Additional file 1: — Main characteristics of cohorts participating in the current CHANCES investigation (Table S1). (DOC 56 kb) [file 12916_2016_607_MOESM1_ESM.doc]

**Table S1** Main characteristics of cohorts participating in the current CHANCES investigation.

| **CHANCES cohort** | **Region covered** | **Follow-up** | | **Mean length [years]** |  | **Participants [N]** | |  | **Total cancer [N]** | |
| --- | --- | --- | --- | --- | --- | --- | --- | --- | --- | --- |
|  |  | **Start** | **End** |  |  | **Total** | **Free of cancer** |  | **Incidence** | **Deaths** |
| COSM | Sweden | 1997-1998 | 2011 | 13 |  | 45906 | 45906 |  | 8335 | 3274 |
| EPIC-Elderly DK | Denmark | 1993-1997 | 2007 | 13 |  | 15355 | 15074 |  | 3161 | 966 |
| EPIC-Elderly ES | Spain | 1992-1996 | 2009 | 13 |  | 5185 | 5026 |  | 782 | 278 |
| EPIC-Elderly GR | Greece | 1994-1999 | 2011 | 10 |  | 9863 | 9492 |  | 826 | 597 |
| EPIC-Elderly NL | Netherlands | 1993-1997 | 2009 | 13 |  | 6896 | 6373 |  | 1093 | 406 |
| ESTHER | Germany | 2000-2002 | 2013 | 11 |  | 9949 | 9278 |  | 1135 | 574 |
| HAPIEE CZ | Czech Republic | 2002-2005 | 2011 | 8 |  | 8857 | n.a. |  | n.a. | 317 |
| HAPIEE LT | Lithuania | 2006-2008 | 2011 | 4 |  | 7161 | n.a. |  | n.a. | 162 |
| HAPIEE PO | Poland | 2002-2005 | 2009 | 7 |  | 10728 | n.a. |  | n.a. | 320 |
| HAPIEE RU | Russia | 2002-2005 | 2010 | 6 |  | 9360 | n.a. |  | n.a. | 226 |
| MORGAM FI | Finland | 1982-2002 | 2010 | 17 |  | 38108 | 37780 |  | 3934 | 1725 |
| MORGAM NI | Northern Ireland | 1991-1994 | 2013 | 16 |  | 2745 | 2721 |  | 494 | 248 |
| MORGAM SE | Sweden | 1986-2009 | 2011 | 11 |  | 5476 | 5247 |  | 459 | 219 |
| NIH-AARP | USA | 1995-1996 | 2008 | 12 |  | 566279 | 557387 |  | 102799 | 36488 |
| RS | Netherlands | 1989-1993 | 2010 | 12 |  | 8121 | 8121 |  | 1729 | 1224 |
| SENECA | Europe | 1988 | 1998 | 8 |  | 2585 | n.a. |  | n.a. | 186 |
| SMC | Sweden | 1987-1990 | 2009 | 13 |  | 38984 | 37265 |  | 5109 | 2441 |
| TROMSØ | Norway | 1994-1995 | 2010 | 13 |  | 10463 | 9818 |  | 2140 | 1146 |
| VIP | Sweden | 1990-1996 | 2007 | 14 |  | 95000 | 95000 |  | 8209 | 2367 |

**Abbreviations** (alphabetically ordered): **COSM**: Cohort Of Swedish Men; **CZ**: Czech Republic; **DK**: Denmark; **EPIC**: European Prospective Investigation into Cancer and Nutrition; **ES**: Spain; **ESTHER**: Epidemiologische Studie zu Chancen der Verhütung, Früherkennung und optimierten Therapie chronischer Erkrankungen in der älteren Bevölkerung [German]; **GR**: Greece; **HAPIEE**: Health, Alcohol and Psychosocial factors In Eastern Europe; **LT**: Lithuania; **MORGAM**: Monica Risk, Genetics, Archiving and Monograph, which included the cohorts **MORGAM FI**: FINRISK Study (Finland); **MORGAM NI**: PRIME Belfast Study (Northern Ireland ); and **MORGAM SE**: Northern Sweden MONICA examinations (Norrbotten county only); **NIH-AARP**: National Institute of Health – American Association of Retired Persons; **NL**: the Netherlands; **PO**: Poland; **RS**: Rotterdam Study; **RU**: Russia; **SENECA**: Survey in Europe on Nutrition and the Elderly a Concerned Action; **SMC**: Swedish Mammography Cohort; **VIP**: Västerbotten Intervention Programme
