# Supplementary material for: Quantification of the smoking-associated cancer risk with rate advancement periods: meta-analysis of individual participant data from cohorts of the CHANCES consortium
Source: BMC Med. 2016 Apr 5;14:62. doi: 10.1186/s12916-016-0607-5 (PMC4820956; doi:10.1186/s12916-016-0607-5)
Supplement: Additional file 2: — Associations of smoking intensity and duration with total and respiratory (DOC 89 kb) [file 12916_2016_607_MOESM2_ESM.doc]

**Table S2** Associations of smoking intensity and duration with total and respiratory tract cancer incidence and mortality. ^1,2^

| **Cancer site** | Smoking exposure | **Cancer incidence** | | | |  | **Cancer mortality** | | | |
| --- | --- | --- | --- | --- | --- | --- | --- | --- | --- | --- |
|  |  | **Total ^3^** | **Cases** | **HR (95% CI)** | **RAP (95% CI)** |  | **Total ^3^** | **Cases** | **HR (95% CI)** | **RAP (95% CI)** |
| **Total cancer** | Smoking intensity in cigs/day ^4^ | | | | |  |  |  |  |  |
|  | Never smoker | 321984 | 43449 | 1.00 (Reference) | 0.00 (Reference) |  | 345173 | 13342 | 1.00 (Reference) | 0.00 (Reference) |
|  | ≤ 9 cig/day | 29267 | 5431 | **1.24 (1.12 ; 1.38)^***^** | **4.41 (2.32 ; 6.49)^***^** |  | 31733 | 2511 | **1.78 (1.50 ; 2.11)^***^** | **6.96 (5.01 ; 8.92)^***^** |
|  | 10-19 cig/day | 44583 | 9476 | **1.45 (1.29 ; 1.64)^***^** | **7.95 (5.45 ; 10.4)^***^** |  | 48685 | 4857 | **2.24 (1.88 ; 2.66)^***^** | **9.88 (7.81 ; 12.0)^***^** |
|  | ≥ 20 cig/day | 37238 | 8833 | **1.69 (1.45 ; 1.97)^***^** | **10.5 (7.34 ; 13.6)^***^** |  | 41878 | 5059 | **2.88 (2.36 ; 3.50)^***^** | **12.8 (10.4 ; 15.1)^***^** |
|  | P linear trend |  |  | **<0.0001** |  |  |  |  | **<0.0001** |  |
|  | Duration of smoking ^4^ | | | | |  |  |  |  |  |
|  | Never smoker | 128221 | 14148 | 1.00 (Reference) | 0.00 (Reference) |  | 150115 | 5684 | 1.00 (Reference) | 0.00 (Reference) |
|  | ≤ 19 years | 35619 | 3532 | 1.14 (0.99 ; 1.31)**^***^** | **2.84 (0.10 ; 5.57)^***^** |  | 39307 | 1100 | 0.96 (0.88 ; 1.04) | -0.30 (-1.25 ; 0.65) |
|  | 20-39 years | 59999 | 8150 | **1.24 (1.09 ; 1.42)^***^** | **5.12 (2.83 ; 7.42)^***^** |  | 71718 | 3623 | **1.48 (1.32 ; 1.66)^**^** | **5.59 ( 4.24 ; 6.95)^**^** |
|  | ≥ 40 years | 30476 | 7098 | **1.63 (1.45 ; 1.84)^***^** | **10.8 (7.80 ; 13.9)^***^** |  | 35772 | 4391 | **2.30 (2.02 ; 2.63)^***^** | **11.3 ( 9.36 ; 13.1)^**^** |
|  | P linear trend |  |  | **<0.0001** |  |  |  |  | **<0.0001** |  |
| **Lung cancer** | Smoking intensity in cigs/day ^4^ | | | | |  |  |  |  |  |
|  | Never smoker | 321984 | 923 | 1.00 (Reference) | 0.00 (Reference) |  | 345173 | 856 | 1.00 (Reference) | 0.00 (Reference) |
|  | ≤ 9 cig/day | 29267 | 971 | **7.52 (5.42 ; 10.4)^**^** | **22.7 (16.2 ; 29.3)^***^** |  | 31733 | 939 | **6.95 (4.86 ; 9.92)^**^** | **19.8 (14.5 ; 25.1)^***^** |
|  | 10-19 cig/day | 44583 | 2354 | **14.0 (10.7 ; 18.4)^**^** | **29.2 (22.4 ; 36.1)^***^** |  | 48685 | 2242 | **12.6 (9.10 ; 17.5)^***^** | **24.9 (19.4 ; 30.5)^***^** |
|  | ≥ 20 cig/day | 37238 | 2721 | **21.7 (17.1 ; 27.4)^**^** | **34.4 (27.5 ; 41.3)^***^** |  | 41878 | 2727 | **18.8 (13.3 ; 26.5)^***^** | **29.6 (23.8 ; 35.5)^***^** |
|  | P linear trend |  |  | **<0.0001** |  |  |  |  | **<0.0001** |  |
|  | Duration of smoking (reference: never smokers) ^4^ | | | | |  |  |  |  |  |
|  | Never smoker | 128221 | 284 | 1.00 (Reference) | 0.00 (Reference) |  | 150115 | 306 | 1.00 (Reference) | 0.00 (Reference) |
|  | ≤ 19 years | 35619 | 99 | **1.67 (1.20 ; 2.34)** | **9.21 (4.51 ; 13.9)** |  | 39307 | 97 | **1.59 (1.16 ; 2.17)** | **7.22 (3.10 ; 11.4)** |
|  | 20-39 years | 59999 | 770 | **5.95 (4.69 ; 7.55)^*^** | **26.9 (21.6 ; 32.1)** |  | 71718 | 813 | **5.02 (3.76 ; 6.69)^**^** | **21.1 (16.2 ; 26.0)** |
|  | ≥ 40 years | 30476 | 1430 | **14.6 (12.1 ; 17.7)** | **40.9 (33.1 ; 48.7)** |  | 35772 | 1597 | **11.9 (9.16 ; 15.6)^**^** | **31.8 (26.9 ; 36.6)** |
|  | P linear trend |  |  | **<0.0001** |  |  |  |  | **<0.0001** |  |
| **Head and neck cancer** | Smoking intensity in cigs/day ^4^ | | | | |  |  |  |  |  |
|  | Never smoker | 321984 | 636 | 1.00 (Reference) | 0.00 (Reference) |  | 345173 | 150 | 1.00 (Reference) | 0.00 (Reference) |
|  | ≤ 9 cig/day | 29267 | 145 | **2.61 (2.14 ; 3.19)** | 6.37 (-5.40 ;18.1)^**^ |  | 31733 | 56 | **4.70 (3.24 ; 6.79)** | 11.1 (-1.28 ; 23.5)^**^ |
|  | 10-19 cig/day | 44583 | 375 | **2.98 (1.93 ; 4.61)^*^** | **15.9 ( 4.83 ; 26.9)^*^** |  | 48685 | 113 | **5.60 (4.18 ; 7.52)** | **14.1 ( 5.99 ; 22.3)** |
|  | ≥ 20 cig/day | 37238 | 474 | **4.74 (3.03 ; 7.42)^*^** | 12.5 (-3.96 ; 29.0)^**^ |  | 41878 | 163 | **8.37 (6.27 ; 11.2)** | **16.5 ( 5.79 ; 27.1)^*^** |
|  | P linear trend |  |  | **<0.0001** |  |  |  |  | **<0.0001** |  |
|  | Duration of smoking ^4^ | | | | |  |  |  |  |  |
|  | Never smoker | 128221 | 177 | 1.00 (Reference) | 0.00 (Reference) |  | 150115 | 61 | 1.00 (Reference) | 0.00 (Reference) |
|  | ≤ 19 years | 35619 | 53 | 1.49 (0.96 ; 2.32) | 1.34 (-4.93 ; 7.60) |  | 39307 | 14 | 1.48 (0.63 ; 3.50) | 1.46 (-9.44 ; 12.4) |
|  | 20-39 years | 59999 | 172 | **2.41 (1.53 ; 3.77)^**^** | **7.07 ( 0.10 ; 14.0)** |  | 71718 | 65 | **2.33 (1,42 ; 3.81)** | **7.01 ( 0.73 ; 13.3)** |
|  | ≥ 40 years | 30476 | 180 | **3.42 (2.37 ; 4.95)** | 1.06 (-8.00 ; 10.1) |  | 35772 | 97 | **3.16 (1.94 ; 5.17)** | 4.43 (-2.98 ; 11.8) |
|  | P linear trend |  |  | **<0.0001** |  |  |  |  | **<0.0001** |  |

^1^ Numbers in bold denote statistical significance (P < 0.05). Heterogeneity was regarded as negligible if not significant (P < 0.05) or I² < 30%. Otherwise, if significant (P < 0.05), it was classified as ^*^ moderate (30% < I² < 50%), ^**^ substantial (50% < I² < 75%), or ^***^ considerable (I² > 75%).

^2^ Cohort-specific Hazard Ratios (HRs) and Rate Advancement Periods (RAPs) were summarized with meta-analyses using random effects models. HRs and RAPs were adjusted for sex, age, BMI, education, vigorous physical activity, history of diabetes and alcohol consumption.

^3^ The total number of participants for the analyses with cancer incidence is smaller because the participants with a diagnosis of cancer before baseline were excluded. Furthermore, HAPIEE and SENECA cohorts had no cancer incidence data available for the analyses.

^4^ Smoking intensity was not available for EPIC-Elderly Sweden and SENECA cohorts. Duration of smoking was not available for NIH-AARP.
