# Supplementary material for: Quantification of the smoking-associated cancer risk with rate advancement periods: meta-analysis of individual participant data from cohorts of the CHANCES consortium
Source: BMC Med. 2016 Apr 5;14:62. doi: 10.1186/s12916-016-0607-5 (PMC4820956; doi:10.1186/s12916-016-0607-5)
Supplement: Additional file 3: — Stratification of meta-analyses and reassessment of heterogeneity for the association of smoking status with total cancer incidence and mortality according to the general cohort characteristics (Table S3). (DOC 82 kb) [file 12916_2016_607_MOESM3_ESM.doc]

**Table S3** Stratification of meta-analyses and reassessment of heterogeneity for the association of smoking status with total cancer incidence and mortality according to the general cohort characteristics. ^1,2^

|  | **Total cancer incidence** | | | | | | | | | |  | **Total cancer mortality** | | | | | | | | | |
| --- | --- | --- | --- | --- | --- | --- | --- | --- | --- | --- | --- | --- | --- | --- | --- | --- | --- | --- | --- | --- | --- |
|  |  | **Former smoking** | | | |  | **Current smoking** | | | |  |  | **Former smoking** | | | |  | **Current smoking** | | | |
|  | **N** | **HR** | **(95%** | **CI)** |  |  | **HR** | **(95%** | **CI)** |  |  | **N** | **HR** | **(95%** | **CI)** |  |  | **HR** | **(95%** | **CI)** |  |
| All countries combined | 14 | **1.15** | **(1.09 -** | **1.21)** | *** |  | **1.42** | **(1.25 -** | **1.62)** | *** |  | 19 | **1.39** | **(1.26 -** | **1.54)** | *** |  | **2.19** | **(1.83 -** | **2.63)** | *** |
| United States | 1 | **1.22** | **(1.20 -** | **1.24)** | n.a. |  | **1.82** | **(1.79 -** | **1.86)** | n.a. |  | 1 | **1.68** | **(1.63 -** | **1.72)** | n.a. |  | **3.51** | **(3.40 -** | **3.63)** | n.a. |
| Europe^3^ | 13 | **1.14** | **(1.08 -** | **1.19)** | ** |  | **1.40** | **(1.30 -** | **1.50)** | *** |  | 18 | **1.37** | **(1.23 -** | **1.52)** | *** |  | **2.13** | **(1.77 -** | **2.56)** | *** |
| Northern | 7 | **1.13** | **(1.07 -** | **1.19)** | ** |  | **1.34** | **(1.25 -** | **1.43)** | ** |  | 7 | **1.40** | **(1.21 -** | **1.63)** | *** |  | **2.38** | **(1.78 -** | **3.17)** | *** |
| Central | 4 | **1.19** | **(1.03 -** | **1.38)** | ** |  | **1.51** | **(1.17 -** | **1.93)** | *** |  | 4 | 1.18 | (0.95 - | 1.47) | *** |  | **1.61** | **(1.09 -** | **2.39)** | *** |
| Southern | 2 | 1.04 | (0.87 - | 1.24) |  |  | **1.50** | **(1.08 -** | **2.07)** | *** |  | 2 | 1.24 | (0.89 - | 1.72) | *** |  | **1.99** | **(1.14 -** | **3.50)** | *** |
| Eastern |  |  | n.a. |  |  |  |  | n.a. |  |  |  | 4 | **1.59** | **(1.22 -** | **2.07)** |  |  | **2.32** | **(1.53 -** | **3.51)** | ** |
| Mean follow-up |  |  |  |  |  |  |  |  |  |  |  |  |  |  |  |  |  |  |  |  |  |
| >12 years | 8 | **1.14** | **(1.07 -** | **1.21)** | ** |  | **1.33** | **(1.21 -** | **1.47)** | *** |  | 8 | **1.27** | **(1.10 -** | **1.47)** | *** |  | **1.85** | **(1.41 -** | **2.43)** | *** |
| ≤ 12 years | 6 | **1.17** | **(1.10 -** | **1.25)** |  |  | **1.53** | **(1.31 -** | **1.80)** | *** |  | 11 | **1.51** | **(1.32 -** | **1.74)** | *** |  | **2.51** | **(1.97 -** | **3.20)** | *** |
| Start year of study |  |  |  |  |  |  |  |  |  |  |  |  |  |  |  |  |  |  |  |  |  |
| Before 1989 | 5 | **1.11** | **(1.06 -** | **1.16)** |  |  | **1.23** | **(1.09 -** | **1.38)** | *** |  | 6 | **1.28** | **(1.07 -** | **1.52)** | *** |  | **2.17** | **(1.58 -** | **2.98)** | *** |
| 1990-1999 | 8 | **1.17** | **(1.08 -** | **1.27)** | *** |  | **1.52** | **(1.28 -** | **1.80)** | *** |  | 8 | **1.38** | **(1.19 -** | **1.60)** | *** |  | **2.07** | **(1.57 -** | **2.72)** | *** |
| 2000 onwards | 1 | **1.21** | **(1.03 -** | **1.41)** | n.a. |  | **1.80** | **(1.51 -** | **2.13)** | n.a. |  | 5 | **1.64** | **(1.31 -** | **2.05)** |  |  | **2.47** | **(1.71 -** | **3.56)** | ** |
| Total number of cases |  |  |  |  |  |  |  |  |  |  |  |  |  |  |  |  |  |  |  |  |  |
| < 2000 | 8 | **1.19** | **(1.08 -** | **1.32)** | ** |  | **1.50** | **(1.31 -** | **1.71)** | ** |  | 15 | **1.41** | **(1.25 -** | **1.60)** | ** |  | **2.19** | **(1.78 -** | **2.69)** | *** |
| ≥ 2000 | 6 | **1.12** | **(1.05 -** | **1.20)** | *** |  | **1.34** | **(1.09 -** | **1.65)** | *** |  | 4 | **1.34** | **(1.11 -** | **1.61)** | *** |  | **2.21** | **(1.53 -** | **3.21)** | *** |

^1^ Numbers in bold denote statistical significance (P < 0.05). Heterogeneity was regarded as negligible if not significant (P < 0.05) or I² < 30%. Otherwise, if significant (P < 0.05), it was classified as ^*^ moderate (30% < I² < 50%), ^**^ substantial (50% < I² < 75%), or ^***^ considerable (I² > 75%).

^2^ Hazard Ratios (HRs) and Rate Advancement Periods (RAPs) adjusted for age, BMI, education, vigorous physical activity, history of diabetes and alcohol consumption.

^3^ Europe was divided into the following regions: Northern: Denmark, Finland, Norway and Sweden; Central: Germany, Netherlands, Northern Ireland; Southern: Greece and Spain.
