# Supplementary material for: Quantification of the smoking-associated cancer risk with rate advancement periods: meta-analysis of individual participant data from cohorts of the CHANCES consortium
Source: BMC Med. 2016 Apr 5;14:62. doi: 10.1186/s12916-016-0607-5 (PMC4820956; doi:10.1186/s12916-016-0607-5)
Supplement: Additional file 4: — Stratification of meta-analyses and reassessment of heterogeneity for the association of smoking status with lung cancer incidence and mortality according to the general cohort characteristics (Table S4). (DOC 79 kb) [file 12916_2016_607_MOESM4_ESM.doc]

**Table** **S4** Stratification of meta-analyses and reassessment of heterogeneity for the association of smoking status with lung cancer incidence and mortality according to the general cohort characteristics. ^1,2^

|  | **Lung cancer incidence** | | | | | | | | | |  | **Lung cancer mortality** | | | | | | | | | |
| --- | --- | --- | --- | --- | --- | --- | --- | --- | --- | --- | --- | --- | --- | --- | --- | --- | --- | --- | --- | --- | --- |
|  |  | **Former smoking** | | | |  | **Current smoking** | | | |  |  | **Former smoking** | | | |  | **Current smoking** | | | |
|  | **N** | **HR** | **(95%** | **CI)** |  |  | **HR** | **(95%** | **CI)** |  |  | **N** | **HR** | **(95%** | **CI)** |  |  | **HR** | **(95%** | **CI)** |  |
| All countries combined | 14 | **4.06** | **(3.13 -** | **5.26)** | ** |  | **13.1** | **(9.90 -** | **17.3)** | *** |  | 19 | **4.10** | **(3.14 -** | **5.36)** | *** |  | **11.5** | **(8.21 -** | **16.1)** | *** |
| United States | 1 | **6.58** | **(6.04 -** | **7.17)** | n.a. |  | **22.9** | **(21.0 -** | **25.0)** | n.a. |  | 1 | **7.21** | **(6.59 -** | **7.89)** | n.a. |  | **24.6** | **(22.4 -** | **27.0)** | n.a. |
| Europe^3^ | 13 | **3.69** | **(3.14 -** | **4.33)** |  |  | **11.8** | **(10.1 -** | **13.6)** |  |  | 18 | **3.87** | **(2.92 -** | **5.13)** | ** |  | **10.8** | **(7.64 -** | **15.4)** | *** |
| Northern | 7 | **3.64** | **(3.03 -** | **4.37)** |  |  | **11.8** | **(10.0 -** | **14.0)** |  |  | 7 | **4.51** | **(2.99 -** | **6.80)** | ** |  | **15.0** | **(8.77 -** | **25.5)** | *** |
| Central | 4 | **4.07** | **(2.56 -** | **6.48)** |  |  | **13.5** | **(8.79 -** | **20.8)** |  |  | 4 | **2.83** | **(1.57 -** | **5.13)** | *** |  | **8.15** | **(3.91 -** | **17.0)** | *** |
| Southern | 2 | **3.46** | **(2.04 -** | **5.88)** |  |  | **8.77** | **(5.11 -** | **15.0)** |  |  | 2 | **4.20** | **(1.80 -** | **9.80)** | *** |  | **9.91** | **(3.47 -** | **28.3)** | *** |
| Eastern | 0 |  | n.a. |  |  |  |  | n.a. |  |  |  | 4 | **3.51** | **(1.70 -** | **7.24)** | *** |  | **8.57** | **(3.78 -** | **19.4)** | *** |
| Mean follow-up |  |  |  |  |  |  |  |  |  |  |  |  |  |  |  |  |  |  |  |  |  |
| >12 years | 8 | **3.69** | **(3.08 -** | **4.42)** |  |  | **11.7** | **(9.93 -** | **13.8)** |  |  | 8 | **4.03** | **(2.73 -** | **5.94)** | *** |  | **11.1** | **(6.76 -** | **18.4)** | *** |
| ≤ 12 years | 6 | **4.39** | **(2.92 -** | **6.61)** | ** |  | **14.4** | **(8.93 -** | **23.3)** | *** |  | 11 | **4.17** | **(2.88 -** | **6.05)** | *** |  | **11.8** | **(7.48 -** | **18.7)** | *** |
| Start year of study |  |  |  |  |  |  |  |  |  |  |  |  |  |  |  |  |  |  |  |  |  |
| Before 1989 | 5 | **3.07** | **(2.44 -** | **3.85)** |  |  | **11.7** | **(9.62 -** | **14.3)** |  |  | 6 | **3.71** | **(2.35 -** | **5.85)** | *** |  | **12.4** | **(6.92 -** | **22.4)** | *** |
| 1990-1999 | 8 | **4.99** | **(3.89 -** | **6.40)** | * |  | **13.5** | **(8.99 -** | **20.3)** | *** |  | 8 | **4.30** | **(2.90 -** | **6.38)** | *** |  | **11.1** | **(6.70 -** | **18.4)** | *** |
| 2000 onwards | 1 | **5.00** | **(2.48 -** | **10.1)** | n.a. |  | **20.0** | **(10.1 -** | **39.4)** | n.a. |  | 5 | **4.38** | **(2.37 -** | **8.10)** | ** |  | **11.0** | **(5.39 -** | **22.5)** | *** |
| Total number of cases |  |  |  |  |  |  |  |  |  |  |  |  |  |  |  |  |  |  |  |  |  |
| < 2000 | 7 | **3.91** | **(2.85 -** | **5.36)** |  |  | **11.7** | **(8.49 -** | **16.2)** |  |  | 11 | **3.70** | **(2.49 -** | **5.52)** | ** |  | **9.1** | **(5.69 -** | **14.7)** | *** |
| ≥ 2000 | 7 | **4.06** | **(2.88 -** | **5.73)** | *** |  | **13.7** | **(9.60 -** | **19.6)** | *** |  | 8 | **4.46** | **(3.11 -** | **6.41)** | *** |  | **14.6** | **(9.01 -** | **23.6)** | *** |

^1^ Numbers in bold denote statistical significance (P < 0.05). Heterogeneity was regarded as negligible if not significant (P < 0.05) or I² < 30%. Otherwise, if significant (P < 0.05), it was classified as ^*^ moderate (30% < I² < 50%), ^**^ substantial (50% < I² < 75%), or ^***^ considerable (I² > 75%).

^2^ Hazard Ratios (HRs) and Rate Advancement Periods (RAPs) adjusted for age, BMI, education, vigorous physical activity, history of diabetes and alcohol consumption.

^3^ Europe was divided into the following regions: Northern: Denmark, Finland, Norway and Sweden; Central: Germany, Netherlands, Northern Ireland; Southern: Greece and Spain.
