# Supplementary material for: Quantification of the smoking-associated cancer risk with rate advancement periods: meta-analysis of individual participant data from cohorts of the CHANCES consortium
Source: BMC Med. 2016 Apr 5;14:62. doi: 10.1186/s12916-016-0607-5 (PMC4820956; doi:10.1186/s12916-016-0607-5)
Supplement: Additional file 5: — Associations of smoking intensity and duration with total and digestive tract cancer incidence and mortality (Table S5). (DOC 88 kb) [file 12916_2016_607_MOESM5_ESM.doc]

**Table** **S5** Associations of smoking intensity and duration with total and digestive tract cancer incidence and mortality. ^1,2^

| **Cancer site** | Smoking exposure | **Cancer incidence** | | | |  | **Cancer mortality** | | | |
| --- | --- | --- | --- | --- | --- | --- | --- | --- | --- | --- |
|  |  | **Total ^3^** | **Cases** | **HR (95% CI)** | **RAP (95% CI)** |  | **Total ^3^** | **Cases** | **HR (95% CI)** | **RAP (95% CI)** |
| **Colorectal cancer** | Smoking intensity in cigs/day ^4^ | | | | |  |  |  |  |  |
|  | Never smoker | 321984 | 4359 | 1.00 (Reference) | 0.00 (Reference) |  | 345173 | 1695 | 1.00 (Reference) | 0.00 (Reference) |
|  | ≤ 9 cig/day | 29267 | 514 | **1.29 (1.17 ; 1.43)** | **3.78 (2.31 ; 5.25)** |  | 31733 | 203 | **1.40 (1.07 ; 1.84)^*^** | **2.86 (0.86 ; 4.87)** |
|  | 10-19 cig/day | 44583 | 762 | **1.30 (1.12 ; 1.52)** | **4.87 (3.64 ; 6.10)** |  | 48685 | 341 | **1.64 (1.36 ; 1.98)** | **5.72 (4.08 ; 7.36)** |
|  | ≥ 20 cig/day | 37238 | 561 | **1.22 (1.10 ; 1.34)** | **3.00 (1.64 ; 4.37)** |  | 41878 | 264 | **1.67 (1.44 ; 1.94)** | **5.85 (4.12 ; 7.57)** |
|  | P linear trend |  |  | **0.0358** |  |  |  |  | **<0.0001** |  |
|  | Duration of smoking ^4^ | | | | |  |  |  |  |  |
|  | Never smoker | 128221 | 1725 | 1.00 (Reference) | 0.00 (Reference) |  | 150115 | 858 | 1.00 (Reference) | 0.00 (Reference) |
|  | ≤ 19 years | 35619 | 450 | **1.22 (1.07 ; 1.40)** | 2.54 (0.88 ; 4.20) |  | 39307 | 164 | 1.05 (0.87 ; 1.28) | 0.40 (-1.78 ; 2.57) |
|  | 20-39 years | 59999 | 956 | **1.23 (1.03 ; 1.48)^**^** | **3.34 (1.35 ; 5.33)** |  | 71718 | 405 | 1.11 (0.95 ; 1.31) | 1.53 (-0.12 ; 3.18) |
|  | ≥ 40 years | 30476 | 760 | **1.25 (1.07 ; 1.46)^*^** | **3.79 (2.22 ; 5.36)** |  | 35772 | 421 | **1.37 (1.18 ; 1.59)** | **3.46 ( 1.63 ; 5.29)** |
|  | P linear trend |  |  | **0.0020** |  |  |  |  | **0.0002** |  |
| **Gastric cancer** | Smoking intensity in cigs/day ^4^ | | | | |  |  |  |  |  |
|  | Never smoker | 321984 | 598 | 1.00 (Reference) | 0.00 (Reference) |  | 345173 | 462 | 1.00 (Reference) | 0.00 (Reference) |
|  | ≤ 9 cig/day | 29267 | 87 | **1.90 (1.46 ; 2.47)** | **6.33 (2.75 ; 9.90)** |  | 31733 | 73 | **2.34 (1.47 ; 3.71)** | **6.03 (2.63 ; 9.43)** |
|  | 10-19 cig/day | 44583 | 132 | **1.80 (1.44; 2.25)** | **6.36 (3.42 ; 9.30)** |  | 48685 | 90 | **1.75 (1.33 ; 2.28)** | **4.30 (1.23 ; 7.38)** |
|  | ≥ 20 cig/day | 37238 | 121 | **1.89 (1.50 ; 2.39)** | **7.53 (4.50 ; 10.6)** |  | 41878 | 95 | **2.10 (1.46 ; 3.01)** | **6.50 (3.78 ; 9.21)** |
|  | P linear trend |  |  | **<0.0001** |  |  |  |  | **<0.0001** |  |
|  | Duration of smoking ^4^ | | | | |  |  |  |  |  |
|  | Never smoker | 128221 | 341 | 1.00 (Reference) | 0.00 (Reference) |  | 150115 | 288 | 1.00 (Reference) | 0.00 (Reference) |
|  | ≤ 19 years | 35619 | 58 | 1.10 (0.79 ; 1.53) | 1.13 (-2.04 ; 4.31) |  | 39307 | 49 | 1.30 (0.86 ; 1.96) | 1.53 (-2.62 ; 5.68) |
|  | 20-39 years | 59999 | 165 | 1.24 (0.93 ; 1.66) | 1.79 (-0.80 ; 4.38) |  | 71718 | 153 | 1.32 (0.96 ; 1.80) | 2.17 (-0.62 ; 4.95) |
|  | ≥ 40 years | 30476 | 177 | **1.64 (1.29 ; 2.08)** | **4.08 ( 1.17 ; 6.99)** |  | 35772 | 169 | **1.69 (1.25 ; 2.29)** | **3.37 ( 0.34 ; 6.40)** |
|  | P linear trend |  |  | **0.0002** |  |  |  |  | **0.0017** |  |
| **Pancreatic cancer** | Smoking intensity in cigs/day ^4^ | | | | |  |  |  |  |  |
|  | Never smoker | 321984 | 921 | 1.00 (Reference) | 0.00 (Reference) |  | 345173 | 1183 | 1.00 (Reference) | 0.00 (Reference) |
|  | ≤ 9 cig/day | 29267 | 156 | **2.21 (1.83 ; 2.67)** | **6.64 (1.31 ; 12.0)^**^** |  | 31733 | 182 | **2.08 (1.74 ; 2.49)** | **8.55 (6.25 ; 10.9)** |
|  | 10-19 cig/day | 44583 | 216 | **2.21 (1.51 ; 3.24)^**^** | **6.42 (0.91 ; 11.9)^**^** |  | 48685 | 282 | **2.99 (2.13 ; 4.20)^**^** | **9.51 (6.45 ; 12.6)** |
|  | ≥ 20 cig/day | 37238 | 186 | **1.93 (1.47 ; 2.53)** | **6.41 (1.57 ; 11.2)^*^** |  | 41878 | 241 | **2.39 (1.79 ; 3.21)** | **9.96 (7.93 ; 12.0)** |
|  | P linear trend |  |  | **<0.0001** |  |  |  |  | **<0.0001** |  |
|  | Duration of smoking ^4^ | | | | |  |  |  |  |  |
|  | Never smoker | 128221 | 344 | 1.00 (Reference) | 0.00 (Reference) |  | 150115 | 461 | 1.00 (Reference) | 0.00 (Reference) |
|  | ≤ 19 years | 35619 | 50 | 0.83 (0.50 ; 1.38) | **-5.88 (-11.4 ; -0.34)** |  | 39307 | 82 | 0.97 (0.62 ; 1.52)^*^ | -2.80 (-6.92 ; 1.32) |
|  | 20-39 years | 59999 | 224 | **1.69 (1.31 ; 2.16)** | **6.92 ( 3.49 ; 10.4)** |  | 71718 | 311 | **1.74 (1.21 ; 2.51)^**^** | **7.78 ( 4.67 ; 10.9)^**^** |
|  | ≥ 40 years | 30476 | 193 | **2.08 (1.56 ; 2.76)** | **7.96 ( 3.98 ; 12.0)** |  | 35772 | 275 | **2.18 (1.68 ; 2.83)** | **8.56 ( 3.25 ; 11.5)^**^** |
|  | P linear trend |  |  | **<0.0001** |  |  |  |  | **<0.0001** |  |

^1^ Numbers in bold denote statistical significance (P < 0.05). Heterogeneity was regarded as negligible if not significant (P < 0.05) or I² < 30%. Otherwise, if significant (P < 0.05), it was classified as ^*^ moderate (30% < I² < 50%), ^**^ substantial (50% < I² < 75%), or ^***^ considerable (I² > 75%).

^2^ Cohort-specific Hazard Ratios (HRs) and Rate Advancement Periods (RAPs) were summarized with meta-analyses using random effects models. HRs and RAPs were adjusted for sex, age, BMI, education, vigorous physical activity, history of diabetes and alcohol consumption.

^3^ The total number of participants for the analyses with cancer incidence is smaller because the participants with a diagnosis of cancer before baseline were excluded. Furthermore, HAPIEE and SENECA cohorts had no cancer incidence data available for the analyses.

^4^ Smoking intensity was not available for EPIC-Elderly Sweden and SENECA cohorts. Duration of smoking was not available for NIH-AARP.
