# Supplementary material for: Quantification of the smoking-associated cancer risk with rate advancement periods: meta-analysis of individual participant data from cohorts of the CHANCES consortium
Source: BMC Med. 2016 Apr 5;14:62. doi: 10.1186/s12916-016-0607-5 (PMC4820956; doi:10.1186/s12916-016-0607-5)
Supplement: Additional file 6: — Associations of smoking intensity and duration with sex-specific cancer incidence and mortality (Table S6). (DOC 70 kb) [file 12916_2016_607_MOESM6_ESM.doc]

**Table** **S6** Associations of smoking intensity and duration with sex-specific cancer incidence and mortality. ^1,2^

| **Cancer site** | Smoking exposure | **Cancer incidence** | | | |  | **Cancer mortality** | | | |
| --- | --- | --- | --- | --- | --- | --- | --- | --- | --- | --- |
|  |  | **Total ^3^** | **Cases** | **HR (95% CI)** | **RAP (95% CI)** |  | **Total ^3^** | **Cases** | **HR (95% CI)** | **RAP (95% CI)** |
| **Breast cancer** | Smoking intensity in cigs/day ^4^ | | | | |  |  |  |  |  |
|  | Never smoker | 174507 | 7121 | 1.00 (Reference) | 0.00 (Reference) |  | 190798 | 1193 | 1.00 (Reference) | 0.00 (Reference) |
|  | ≤ 9 cig/day | 15852 | 697 | 1.03 (0.90 ; 1.18) | 3.34 (-0.68 ; 7.37) |  | 17302 | 117 | 1.17 (0.94 ; 1.45) | 2.63 (-1.86 ; 7.12) |
|  | 10-19 cig/day | 22766 | 1020 | **1.10 (1.00 ; 1.20)** | **3.07 ( 0.00 ; 6.14)** |  | 24079 | 185 | **1.34 (1.01 ; 1.77)** | **4.28 ( 0.63 ; 7.92)** |
|  | ≥ 20 cig/day | 12664 | 538 | 1.05 (0.95 ; 1.15) | 1.53 (-2.63 ; 5.70) |  | 13760 | 118 | **1.66 (1.18 ; 2.33)** | **8.67 ( 4.01 ; 13.3)** |
|  | P linear trend |  |  | 0.5563 |  |  |  |  | **0.0024** |  |
|  | Duration of smoking ^4^ | | | | |  |  |  |  |  |
|  | Never smoker | 77111 | 2495 | 1.00 (Reference) | 0.00 (Reference) |  | 93360 | 493 | 1.00 (Reference) | 0.00 (Reference) |
|  | ≤ 19 years | 16134 | 485 | 1.13 (0.92 ; 1.40)^**^ | -1.55 (-5.58 ; 2.48) |  | 17826 | 75 | 1.35 (0.87 ; 2.10) | 1.92 (-3.40 ; 7.24) |
|  | 20-39 years | 26246 | 987 | **1.22 (0.98 ; 1.52)^***^** | 1.49 (-1.75 ; 4.73) |  | 31017 | 180 | 1.21 (0.98 ; 1.50) | 3.61 (-0.16 ; 7.38) |
|  | ≥ 40 years | 11068 | 427 | 1.08 (0.96 ; 1.22) | -0.59 (-6.02 ; 4.83) |  | 12344 | 93 | 1.39 (0.93 ; 2.09) | -0.03 (-6.96 ; 6.89) |
|  | P linear trend |  |  | 0.0710 |  |  |  |  | **0.0465** |  |
| **Prostate cancer** | Smoking intensity in cigs/day ^4^ | | | | |  |  |  |  |  |
|  | Never smoker | 147477 | 11090 | 1.00 (Reference) | 0.00 (Reference) |  | 154375 | 911 | 1.00 (Reference) | 0.00 (Reference) |
|  | ≤ 9 cig/day | 13415 | 828 | **0.87 (0.80 ; 0.93)** | **-2.25 (-3.58 ; -0.91)** |  | 14431 | 145 | **1.65 (1.34 ; 2.02)** | **3.41 (1.96 ; 4.86)** |
|  | 10-19 cig/day | 21817 | 1286 | **0.86 (0.81 ; 0.92)** | **-2.33 (-3.40 ; -1.26)** |  | 24070 | 207 | **1.79 (1.51 ; 2.13)** | **4.05 (2.88 ; 5.23)** |
|  | ≥ 20 cig/day | 24574 | 1126 | **0.76 (0.63 ; 0.92)^**^** | **-3.66 (-6.28 ; -1.03)^*^** |  | 28118 | 154 | **1.49 (1.14 ; 1.93)** | **2.88 (1.59 ; 4.17)** |
|  | P linear trend |  |  | **0.0011** |  |  |  |  | **0.0001** |  |
|  | Duration of smoking ^4^ | | | | |  |  |  |  |  |
|  | Never smoker | 51110 | 2758 | 1.00 (Reference) | 0.00 (Reference) |  | 56755 | 425 | 1.00 (Reference) | 0.00 (Reference) |
|  | ≤ 19 years | 19485 | 974 | 0.98 (0.85 ; 1.11) | 0.12 (-0.82 ; 1.07) |  | 21481 | 129 | 1.09 (0.87 ; 1.38) | 0.41 (-1.24 ; 2.05) |
|  | 20-39 years | 33753 | 1740 | 0.86 (0.72 ; 1.01)^**^ | -0.81 (-2.86 ; 1.24)^**^ |  | 40701 | 269 | 1.02 (0.85 ; 1.22) | 0.38 (-0.85 ; 1.62) |
|  | ≥ 40 years | 19408 | 1334 | **0.88 (0.81 ; 0.97)** | **-1.59 (-2.64; -0.55)** |  | 23428 | 366 | 1.16 (0.92 ; 1.46) | 0.57 (-0.73 ; 1.86) |
|  | P linear trend |  |  | 0.1292 |  |  |  |  | 0.1978 |  |

^1^ Numbers in bold denote statistical significance (P < 0.05). Heterogeneity was regarded as negligible if not significant (P < 0.05) or I² < 30%. Otherwise, if significant (P < 0.05), it was classified as ^*^ moderate (30% < I² < 50%), ^**^ substantial (50% < I² < 75%), or ^***^ considerable (I² > 75%).

^2^ Cohort-specific Hazard Ratios (HRs) and Rate Advancement Periods (RAPs) were summarized with meta-analyses using random effects models. HRs and RAPs were adjusted for sex, age, BMI, education, vigorous physical activity, history of diabetes and alcohol consumption.

^3^ The total number of participants for the analyses with cancer incidence is smaller because the participants with a diagnosis of cancer before baseline were excluded. Furthermore, HAPIEE and SENECA cohorts had no cancer incidence data available for the analyses.

^4^ Smoking intensity was not available for EPIC-Elderly Sweden and SENECA cohorts. Duration of smoking was not available for NIH-AARP.
